# DDX17-Mediated Upregulation of CXCL8 Promotes Hepatocellular Carcinoma Progression *via* Co-activating β-catenin/NF-κB Complex

**DOI:** 10.7150/ijbs.104165

**Published:** 2025-01-20

**Authors:** Hui Liu, Xiaoliang Gao, Wenyao Zhang, Xin Fu, Jing Zhang, Qiangqiang Yuan, Jing Jin, Xinyu Du, Renlong Li, Yan Li, Jun Yu, Qiujin Zhang, Xianchun Gao, Liang Zhang, Yuwei Ling, Jing Wu, Lin Wang, Jinliang Xing, Fulin Chen, Yongzhan Nie

**Affiliations:** 1 State Key Laboratory of Holistic Integrative Management of Gastrointestinal Cancers and National Clinical Research Center for Digestive Diseases, Xijing Hospital of Digestive Diseases, Fourth Military Medical University, Xi'an, China.; 2 Key Laboratory of Resource Biology and Biotechnology in Western China, Ministry of Education. School of Medicine, Northwest University, Xi'an, China.; 3 Department of Hepatobiliary Surgery, Xijing Hospital, Fourth Military Medical University, Xi'an, China.; 4 Institute of Analytical Chemistry and Instrument for Life Science, The Key Laboratory of Biomedical Information Engineering of Ministry of Education, School of Life Science and Technology, Xi'an Jiaotong University, Xi'an, China.; 5 State Key Laboratory of Holistic Integrative Management of Gastrointestinal Cancers, Department of Physiology and Pathophysiology, Fourth Military Medical University, Xi'an, China.

**Keywords:** DEAD-box RNA helicase 17, C-X-C motif chemokine ligand 8, Hepatocellular carcinoma, NF-κB, CXCR1/2 inhibitor

## Abstract

Hepatocellular carcinoma (HCC) is a well-known inflammation-related cancer, that accounts for fifth most prevalent neoplasm and the third major driver of cancer associated fatality globally. Accumulating evidence has elucidated that C-X-C motif chemokine ligands (CXCLs) are aberrantly upregulated in HCC and are involved in inflammation-induced hepatocarcinogenesis and metastasis. Herein, we identified a novel function of DEAD-box RNA helicase 17 (DDX17) as an oncogenic factor via transactivating CXCL8 in HCC. Unlike the adjacent nontumor tissues, DDX17 was highly expressed in tumor tissues compared in two independent cohorts and that it acts as an independent prognostic indicator for patients who have HCC. Mechanistically, DDX17 interacts with β-catenin and NF-κB, and promotes their nuclear translocation to promote the transcription of the inflammatory gene CXCL8, thus promoting HCC proliferation and invasion *in vitro* and *in vivo*. More interestingly, stimulation with recombinant human CXCL8 augmented the interaction of NF-κB with DDX17/β-catenin and enhanced its autocrine activation by promoting the phosphorylation of IκBα. Furthermore, blocking the association of the DDX17/β-catenin/NF-κB complex with a CXCR1/2 inhibitor markedly abrogated DDX17-mediated HCC proliferation and metastasis. Overall, this study provided new insights into DDX17-mediated pro-inflammatory chemokine activation, which unveiled the association between DDX17 and β-catenin/ NF-κB complex in transactivating the expression of CXCL8. The usage of CXCR1/2 inhibitor to block DDX17-induced CXCL8 signaling activation might be a potential therapeutic approach for HCC treatment.

## Introduction

On a global scale, hepatocellular carcinoma (HCC) is the fifth most prevalent neoplasm and the third major driver of cancer associated fatality 1. With an incidence of approximately 500,000 new cases per year, it is estimated that one million individuals will suffer from this lethal illness by 2030 2, 3, which imposes a considerable global health burden. Regrettably, the underlying molecular mechanisms contributing to the progression of HCC and its highly invasive nature remain elusive. Hence, studies focused on elucidating the molecular mechanism underlying HCC progression and identifying reliable targets to predict therapeutic response are urgently needed.

Notably, HCC is well known as an inflammation-related cancer. Chronic liver injury derived from hepatitis virus infection, metabolic dysfunction, or other pathogenic factors, is responsible for inflammation exacerbation and hepatocellular carcinogenesis, which results in the excessive secretion of a variety of cytokines and chemokines 4. The C-X-C motif chemokine ligands (CXCLs) have been elucidated to be aberrantly upregulated in HCC and promote cancer progression by favoring cancer cell proliferation and invasion, as well as recruiting immunosuppressive cells 5-7. CXCL8, alternatively referred to as interleukin-8 (IL-8), is a canonical proinflammatory and prometastatic chemokine involved in multiple stages of tumor progression 8. Studies have indicated that the upregulation of CXCL8 both in serum and in cancerous lesions is associated with poor prognosis and malignant behavior of HCC cells 9-11. Typically, CXCL8 gene expression was activated by the transcriptional factor NF-κB via nuclear translocation 12. Nevertheless, the molecular mechanism through which CXCL8 was regulated remains poorly understood, and figuring out what underlying mechanism might be involved in NF-κB induced CXCL8 expression will provide a better understanding of HCC.

DEAD-box RNA helicase family has been renowned for its functions in RNA metabolism, encompassing pre-mRNA processing, alternative splicing, and miRNA biogenesis 13. Among them, DDX17 is well-characterized as a co-factor of the microprocessor, which controls miRNA recognition and maturation. Importantly, DDX17 has drawn much attention for its critical role as a transcriptional co-factor in lung cancer, breast cancer, and colorectal cancer initiation and development 14-16. In HCC, DDX17 was found to be up-regulated in a large number of patients 17, 18. It has been reported that DDX17 overexpression was closely related to the stemness remodeling in stem cells and the malignant progression of HCC 19. In particular, approximately 80% to 90% of HCC patients are precipitated by hepatitis viral infections, non-alcoholic steatohepatitis, and other factors which may lead to chronic inflammation and cirrhosis of the liver 20. DDX17 was reported to promote HBV-related HCC development by facilitating HBV transcription and replication 21. Another recent study indicated the critical role of DDX17 in contributing to inflammatory gene expression in murine non-alcoholic steatohepatitis (NASH) model. DDX17 overexpression mediated the activation of M1 macrophages and increased the expression of proinflammatory cytokines such as TNF-ɑ, IL-6, CCL2, thereby exacerbating liver steatosis and fibrosis 22. However, to our knowledge, the oncogenic role of DDX17 in HCC progression has not yet been fully elucidated. Particularly, the underlying mechanism through which DDX17 regulates inflammatory signaling activation in HCC, a prototypical inflammation-related cancer, has not been investigated.

Herein, we found that DDX17 is highly expressed and presents as an independent prognostic factor in HCC. DDX17 overexpression promoted tumor growth and enhanced the migrative and invasive capabilities of HCC cells. Mechanistically, DDX17 coupled with the β-catenin/ NF-κB complex and promoted their nuclear translocation to activate the transcription of the chemotactic gene *CXCL8*. More importantly, CXCL8 augmented the interaction of NF-κB with DDX17/β-catenin and enhanced its autocrine activation via phosphorylating NF-κB/p65. Inhibition of CXCR1/2, the transmembrane receptor of CXCL8, remarkably abrogated DDX17-mediated HCC growth and metastasis *in vitro* and *in vivo*. Taken together, our findings offered new insights into DDX17-mediated pro-inflammatory chemokine activation, which unveiled the association between DDX17 and β-catenin/ NF-κB complex in transactivating the expression of CXCL8. The usage of CXCR1/2 inhibitor to block DDX17-induced CXCL8 signaling activation might be a potential therapeutic approach for HCC management.

## Materials and Methods

### Cell culture

HCC cells (HepG2, Huh7, SK-hep1, MHCC97H, HCCLM3, HCCLM6, and Hep3B; the Institute of Biochemistry and Cell Biology, Chinese Academy of Science, China) were cultured in DMEM containing 10% FBS (Thermo Fisher Scientific, USA), penicillin (100 μg/mL), and streptomycin (100 U/mL) at 37 °C in the incubator supplemented with 5% CO2. For CXCR1/2 blockade, cells were administered with Reparixin at the concentration of 1 μM. Cell lines were authenticated through short tandem repeat (STR) DNA profiling, and confirmed to be free of mycoplasma contamination.

### Lentivirus infection and small interfering RNA (siRNA) transfection

To execute lentivirus infection, we added 1 ml of lentivirus working solution to each well of a 6-well plate seeded with HCC cells (HepG2, Huh7, and SK-hep1) based on virus titer and multiplicity of infection of cells. Then, the cells were incubated for 12 h before replacing them with a complete DMEM. Then, 2.5 µg/mL puromycin was introduced to select effective transfected cells following incubation for 72 h. Genechem (Shanghai, China) constructed The DDX17-knockdown and overexpression lentivirus. Briefly, the full-length cDNAs of human DDX17 were cloned and inserted into the GV341 vector, namely, Ubi-MCS-3FLAG-SV40-puromycin. The short hairpin RNA (shRNA) targeting DDX17 was subjected to cloning into the GV112 vector, described as hU6-MCS-CMV-puromycin. shRNA targeting sequence was described as 5'-GCAUCUGACCCGUGCUAUU-3'.

For siRNA transfection, HCC cells were planted in a 6-well plate. After one day, the siRNAs (GenePharma, China) were transfected into these cells with HiPerFect Transfection Reagent (QIAGEN, German) per the protocols, assessing the transfection efficiency by RT-qPCR or Western blotting. Table S1 lists siRNA targeting sequences.

### RNA extraction and quantitative Real-time PCR (RT-qPCR)

Following the protocols, cell/tissue total RNA was extracted by the RNeasy Mini Kit (Qiagen, Germany), thereby reversely transcribing RNA into cDNA with the the Prime Script RT Reagent Kit (TaKaRa, Japan). RT-PCR was performed in triplicate using SYBR Premix Ex TaqTM II (Yeasen, China), with the CFX96 QPCR Detection System (Bio-Rad, USA). Table S2 summarizes the primer sequences.

### Protein extraction and western blotting

The extraction of cell nuclear and cytoplasmic protein was performed via Nuclear and Cytoplasmic Protein Extraction Kit (#P0028, Beyotime, China) per protocols. Total protein was extracted from cells or tissue using RIPA lysis buffer (Beyotime, China), which was supplemented with 1x phosphatase and 1x protease inhibitors (Roch, Sigma Aldrich). After centrifuging at 12000 g, at 4°C for 15 min, the supernatants carefully were transferred into 1.5 ml tube and total protein concentration was quantified by a BCA Protein Assay Kit. Proteins extracted from lysed cells were fractionated via SDS-PAGE, subsequently transferred to nitrocellulose membranes (Millipore), and incubated overnight. Following this, the membranes were re-incubated with HRP-conjugated secondary anti-mouse/rabbit antibody at room temperature for 1 h. The chemiluminescent signal visualization was performed by an Immobilon Western HRP Substrate Kit (Millipore, USA). Table S3 lists the information on primary antibodies. Anti-β-actin served as the control.

### Immunofluorescence (IF) assay

The cells were exposed to 4% paraformaldehyde for 15 min, and permeabilized with 0.5% Triton X-100 for 15 minutes. After blocking with 5% goat serum at 37 °C for 30 min, the cells were incubated with primary antibodies at 4 °C overnight. Following staining with the corresponding fluorescent secondary antibodies for 30 min, DAPI was deployed for nuclei counterstaining for another 5 min. The images were acquired using the Nikon A1 Confocal Laser Microscope System. The anti-β-catenin (1:100, #8480; CST) and anti-p65 (1:50, T55034M, Abmart) were used for the IF assay.

### Cell proliferation assay

To determine the cell viability, we seeded HCC cells into 96-well plates at 1 × 10^4^ cells per well, and incubated them with a CCK-8 solution for another 2 h at 1, 2, 3, and 4 days. The absorbance of each well was measured at 450 nm by VARIOSKAN FLASH (Thermo Fisher Scientific).

The thymidine analog 5-ethynyl-2'-deoxyuridine (EdU) assay was performed by seeding HCC cells to 24-well plates, thereby utilizing an EdU assay kit (RiboBio) to detect the positive cells in line per protocols. The images were acquired using the Nikon A1 Confocal Laser Microscope System and the ratio of positive cells was quantified by Image J software.

For colony formation assay, we seeded HCC cells into a 6-well plate at 2 × 10^3^ cells per well, and cultured cells for 2 weeks with a complete medium until colonies became visible. After fixation with ethanol for 5 min, staining with 0.5% crystal violet for another 5 min, the cells were photographed and quantified.

### Transwell and invasion assay

Initially, 5×10^5^ HCC cells suspended with 200 μL of serum-free medium were introduced to the upper chamber of the inserts, and 700 μL DMEM that contained 20% FBS was introduced into the lower chamber. Following incubation for 16-36 hours, the cells were rinsed with PBS, fixed with 4% formaldehyde for 30 min, and subsequently stained with 0.1% crystal violet solution for 30 min. After washing with PBS, cells remaining on the upper chamber were gently wiped using the cotton swab. Furthermore, the average number of migrated cells was quantified by three random fields from each of three independent inserts.

### Co-immunoprecipitation (Co-IP)

Following the protocols, Co-IP was performed through a Pierce Co-IP Kit (Thermo Fisher Scientific, USA). The extracts were exposed to overnight incubation with anti-DDX17 (#657302, Biolegend), anti-β-catenin (#8480, CST), and anti-p65 (#8242, CST) antibodies for endogenous antigen detection, while the primary antibody anti-HA (sc-53516, SANTA) was used for exogenous DDX17 immunoprecipitation. Isotype mouse or rabbit immunoglobulin G (Beyotime) served as the negative control.

### *In situ* proximity ligation assay (PLA)

Mouse anti-DDX17 antibody (1:100, #657302, BioLegend), rabbit anti-β-catenin antibody (1:100, #8480; CST) and rabbit anti-p65 antibody (1:50, T55034M, Abmart) were used as primary antibodies owing to their specificities in IF assays. Duolink *In Situ* Red Starter Kit Mouse/Rabbit (Millipore-Sigma, #DUO92101) was employed and the procedure was executed in line with the standard protocol 23. Briefly, cells were seeded in eight-well fluorescent chambers at a confluency of 20-30% one day prior to the experiment. After cells adhered to the wall, they were fixed, permeabilized and blocked. Different primary antibodies were employed to detect physical interaction between two proteins. Negative controls included the single-stained DDX17 group and the DDX17 plus IgG group, while the DDX17-DXX17 group acted as the positive control. PLA probes (anti-rabbit Plus and anti-mouse Minus) were utilized as secondary antibodies. Under the action of ligase, the oligonucleotides were brought into proximity with each other to form a closed circle. Under the action of amplification enzyme, rolling circle replication continuously formed new closed circles, generating rolling circle amplification with red fluorescence. Finally, Duolink Antifade Mounting Medium containing DAPI was utilized for sealing. The images were acquired using the Confocal Laser Scanning Microscope System.

### Dual luciferase assay

The dual-luciferase assay was performed as described before 24. Concisely we seeded 1×10^4^ cells into a 96-well plate, a reporter plasmid carrying CXCL8 promoter, a human DDX17 plasmid, along with p65 siRNA, was co-transfected into cells with Lipofectamine 2000 (Invitrogen USA, #11668030). Per protocols, luciferase intensity was measured via Dual-Luciferase Reporter Assay (Promega, USA). The relative luciferase activity was determined by Firefly/Renilla luciferase intensity.

### ELISA assay

A human CXCL8 ELISA Kit (Dakewe, China) was utilized to determine CXCL8 concentration in the supernatant of the cell. The samples and washing buffer were added to each well in line with the protocols. The absorbance of each well was measured at 450 nm via a Thermo Fisher Scientific Varioskan Flash multimode reader.

### Tissue specimens and immunohistochemistry (IHC) assay

Commercial tissue microarrays (TMAs), including HLivH180Sull and HEsoS180, were purchased from Shanghai Outdo Biotech Company (https://product.superchip.com.cn:83). Here, 80 pairs of HCC and adjacent normal tissues, along with an additional 8 cases of HCC samples, were authenticated because some dots were detached during the antigen retrieval. The ESCC microarray includes 90 cases of cancer samples and 68 cases of adjacent normal samples. In addition, 15 cases of paired HCC and adjacent normal tissues used for detecting DDX17 mRNA and protein levels were acquired from the Department of Hepatobiliary Surgery, Xijing Hospital under Ethics Committee approval, with all participants signing informed consent. This study conforms to the Declaration of Helsinki. The workflow of IHC staining was implemented as described previously 25. Anti-DDX17 (1:100, ab180190; Abcam) antibody and 3,3-diaminobenzidine (DAB) kits (Vector Laboratories, Peterborough, UK) were deployed for IHC assay. Herein, 1 (0-25%), 2 (25-50%), 3 (50-75%) and 4 (> 70%) indicated the positive cell percentage, while 0 (no staining), 1 (weak staining), 2 (moderate staining) or 3 (strong staining) indicating staining intensity. The final score was calculated as the positive cell percentage × staining intensity score. If the final score was ≤ 6, the DDX17 expression was defined as “low expression”. Meanwhile, a score of > 6 was considered as “high expression”.

### Animal study

Six-week-old BALB/c nude mice were obtained from the Beijing Vital River Experimental Animal Center. Herein, 4 × 10^6^ the indicated cells were slowly administered into the caudal vein or posterior flanks of the mice. The subcutaneous tumor volumes were determined at every week. For CXCR1/2 inhibition *in vivo*, Reparixin was intraperitoneally injected into the mice at 5 mg/kg with three times weekly. After the mice were euthanized, tissues were embedded and stained with H&E or the indicated antibodies at the terminal point. Animal experiments were authorized and supervised by the Animal Care and Use Committee of the Air Force Military Medical University.

### Statistical analysis

Experiments were executed at least three times to ensure reproducibility. Data were analyzed through SPSS 22.0 or GraphPad Prism 9.0, and presented as mean ± SD. Student's t-test was deployed to determine the differences between two independent groups. The Mann-Whitney *U* test was utilized, aiming at comparing the differences between metastatic lung nodules when the variances differed. Growth curves were analyzed via Two-way ANOVA with repeated measurements. Differences of IHC scores between cancerous and adjacent normal tissues were determined by Wilcoxon's matched-pairs signed-rank test. Kaplan‒Meier analysis was employed to evaluate overall survival and univariate and multivariate analyses were determined by a Cox proportional hazards model. *P* < 0.05 indicated statistically significant. *p < 0.05, **p < 0.01, ***p < 0.001.

## Results

### DDX17 is significantly upregulated and correlates with poor survival in HCC patients

**Figure 1:**
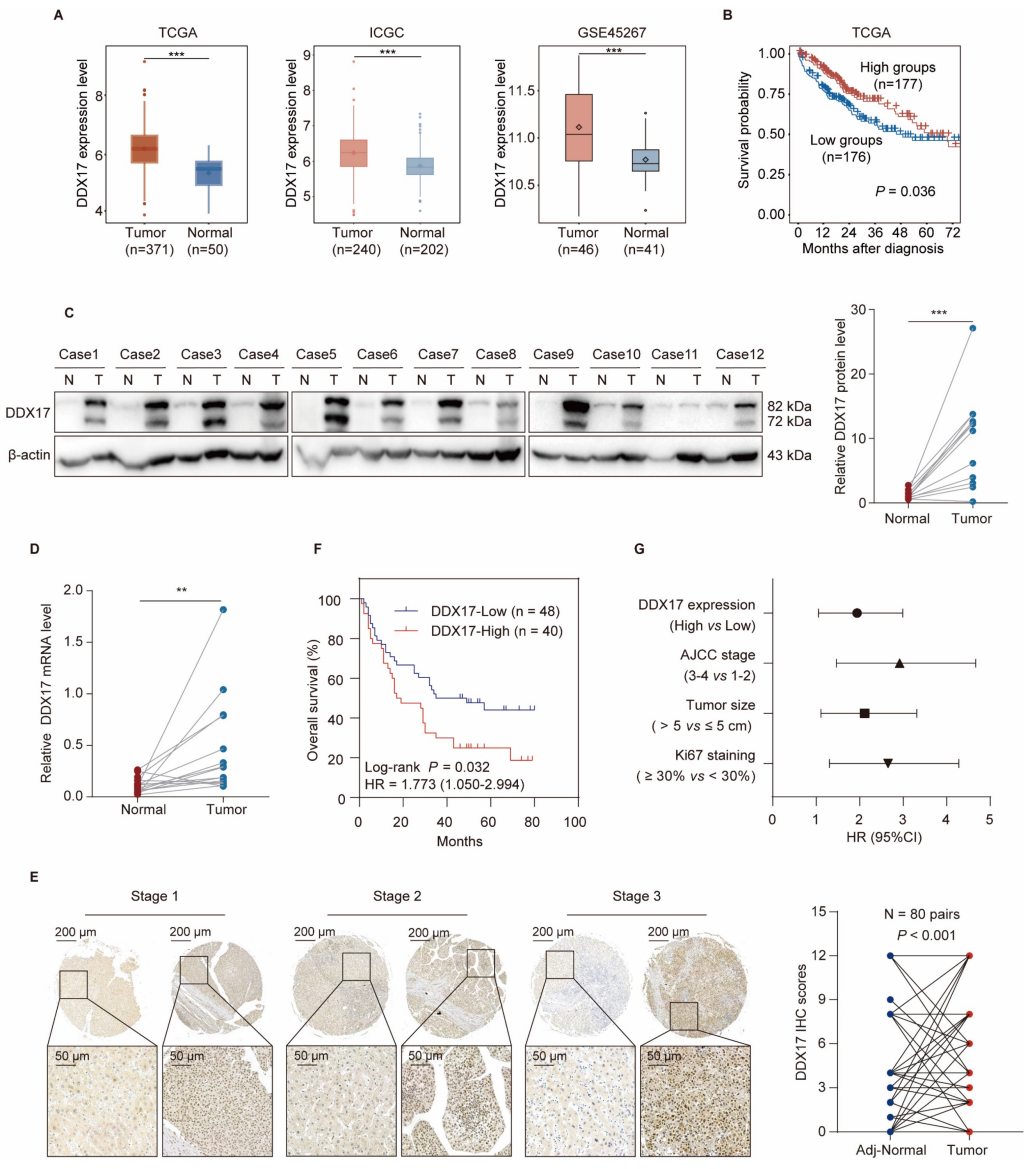
** DDX17 is significantly upregulated and correlates with poor survival in HCC patients. (A)** DDX17 expression in HCC and normal tissues in the TCGA, ICGC and GSE45267 databases. **(B)** Kaplan‒Meier analysis was deployed to evaluate the correlation between DDX17 and overall survival (OS) in TCGA. Categorization of HCC patients with more than 15 days of survival into low DDX17 and high DDX17 expression groups.** (C)** Western blotting was used to detect DDX17 protein levels in primary HCC and adjacent normal specimens (n = 12).** (D)** RT-qPCR measured the DDX17 mRNA levels in HCC and adjacent normal specimens (n = 15). **(E)** Representative micrographs of IHC staining for DDX17 in HCC and adjacent normal tissues. Differences of IHC scores between HCC and adjacent normal tissues (n = 80 pairs) were determined by Wilcoxon's matched-pairs signed-rank test. Scale bars, 200 µm (upper), 50 µm (lower).** (F)** Kaplan‒Meier analysis of the correlation between DDX17 expression and OS in 88 HCC patients.** (G)** Univariate Cox regression analysis was used to assess the association between clinicopathological factors and overall survival. **p < 0.01, ***p < 0.001.

Accumulated evidence has demonstrated that DDX17 is dysregulated in diverse types of cancers. To determine whether DDX17 is upregulated in human HCC tissues, we first compared DDX17 mRNA levels of DDX17 between HCC and normal tissues in different online databases, including the Cancer Genome Atlas (TCGA), International Cancer Genome Consortium (ICGC) and Gene Expression Omnibus (GEO; GSE45267). Results showed that DDX17 mRNA was significantly overexpressed in HCC tissues in contrast to the normal group (Fig. 1A). Additionally, correlation between DDX17 expression and HCC patient survival was further evaluated, revealing that higher DDX17 expression indicated an inferior prognosis (Fig. 1B). Fifteen cases of HCC and unpaired adjacent non-tumorous tissues were analyzed. The DDX17 mRNA and protein levels were significantly upregulated in tumor tissues in contrast to the normal group (Fig. 1C, D).

To further explicit the expression pattern of DDX17, a tissue microarray comprising 80 pairs of HCC and adjacent normal tissues, along with an additional 8 cases of HCC samples, was utilized. The IHC results indicated that DDX17 mostly located in the nucleus of tumor cells, and DDX17 expression progressively increased across different pathological stages of HCC, unlike the adjacent normal tissues (Fig. 1E). Kaplan-Meier analysis manifested those patients with high DDX17 expression indicated a shorter OS in contrast to those with low DDX17 expression (*P* = 0.028) (Fig. 1F). The correlation between DDX17 expression and the clinicopathological characteristics of HCC patients was evaluated, and results revealed that DDX17 expression was positively correlated with patient age, tumor histological stage, and Ki67 and PD-L1 staining (Table 1). Univariate and multivariate Cox analyses revealed that DDX17 expression was an independent prognostic factor for HCC patients (Fig. 1G; Table 2).

To investigate the causal relationship between DDX17 expression and prognosis in additional cancer types of the digestive system. An independent esophageal squamous cell carcinoma (ESCC) cohort containing 90 cases of ESCC tissues and 68 cases of adjacent normal tissues was employed. Compared to the levels in normal tissues, DDX17 expression gradually increased with the advancement of pathological stages (Fig. S1A, B). Furthermore, high DDX17 expression was related to shorter OS in patients who have ESCC (Fig. S1C). DDX17 expression was positively correlated with the *N* stage and AJCC stage (Table S4). Univariate and multivariate Cox regression analyses indicated that DDX17 expression would serve as an independent prognostic factor for ESCC patients (Fig. S1D; Table S5). Collectively, DDX17 expression was significantly elevated in multiple digestive system tumors and positively correlated with poor survival.

### DDX17 enhances HCC growth, migration, and invasion *in vitro* and* in vivo*

**Figure 2:**
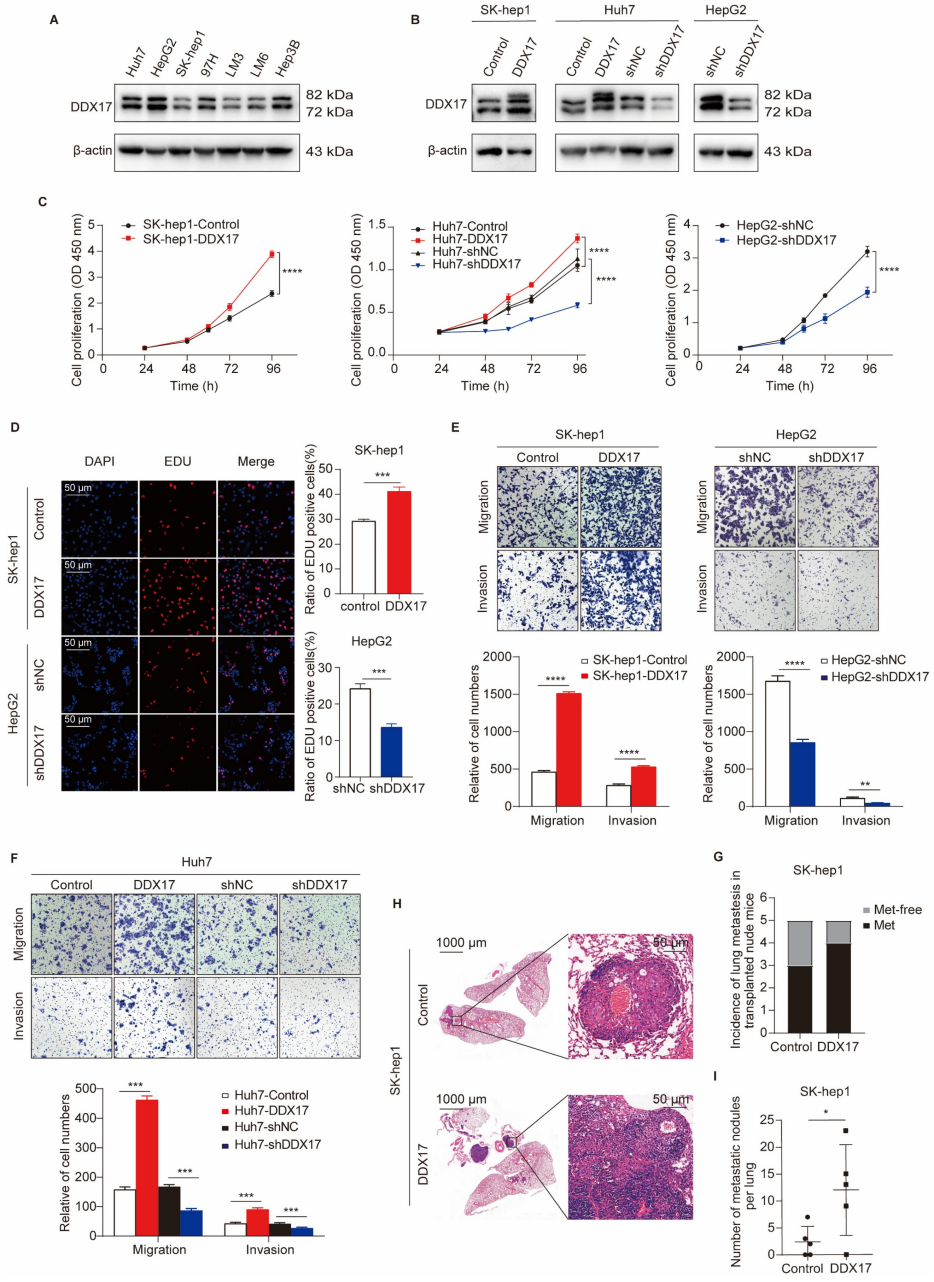
** DDX17 promotes HCC proliferation, migration, and invasion *in vitro* and* in vivo.* (A)** Western blotting was utilized to detect DDX17 protein levels in seven types of HCC cell lines, including Huh7, HepG2, SK-hep1, MHCC97H, HCCLM3, HCCLM6, and Hep3B. **(B)** The efficiency of overexpression or knockdown by lentiviral transduction was determined by Western blotting. **(C)** CCK8 assay detected the proliferation capacities of the indicated HCC cells (n = 3). **(D)** EdU assays detected the effect of DDX17 overexpression or knockdown on cell proliferation (Scale bars, 50 µm; n = 3).** (E-F)** Transwell and Invasion assays measured indicated HCC cell migratory and invasion after DDX17 knockdown or ectopic expression (n = 3).** (G)** Injection of SK-hep-1 cells transfected with empty vector or DDX17 overexpression into the caudal vein of nude mice. Lung metastasis incidence of lung in the indicated group was measured before euthanasia (n = 5). **(H)** Representative H&E staining of the lung tissues in the indicated nude mice (Scale bars, 1000 µm for the left, 50 µm for the right). **(I)** Number of metastatic nodules per lung in the indicated group was counted. The Mann-Whitney *U* test was applied to compare the disparities between two groups. *p < 0.05, **p < 0.01, ***p < 0.001.

Previous studies have indicated DDX17 oncogenic implications in gastrointestinal tumors 16, 26. To investigate its potential role in HCC, we first ascertained DDX17 protein levels in seven diverse HCC cell lines obtained from the ATCC. Western blot analysis manifested that DDX17 was relatively lower expressed in SK-hep1 and HCCLM3, while relatively higher expressed in HepG2. Subsequently, lentivirus was used to stably overexpress or knockdown DDX17 in the investigated cells, which was validated by Western blot and RT-qPCR (Fig. 2A, B; Fig. S2A, B). As shown in Fig. 2C, DDX17 ectopic expression increased the cell viability of SK-hep1 and Huh7, while DDX17 knockdown inhibited cell viability in HepG2 and Huh7 cells. Consistently, DDX17's effects on cell proliferation were further validated via EdU assays (Fig. 2D; Fig. S2C). To determine the pro-metastatic effect of DDX17 on HCC cells, migration and Matrigel invasion assays were performed. DDX17 overexpression significantly escalated HCC migration and invasion, whereas its knockdown significantly suppressed Huh7 and HepG2 migration and invasion (Fig. 2E, F). To explore the potential pro-metastatic effect *in vivo*, indicated cells with stable DDX17 overexpression were intravenously injected into the nude mice via the tail vein. DDX17 overexpression significantly increased the incidence of lung metastasis compared to the control (Fig. 2G). Furthermore, the number of metastatic nodules also increased in the SK-hep1-DDX17 group, as evidenced by H&E staining (Fig. 2H, I). Collectively, these results suggested that DDX17 functioned as an oncogenic role in HCC by enhancing cell growth, migration, and invasion.

### DDX17 promotes HCC progression through mediating β-catenin nuclear translocation

**Figure 3:**
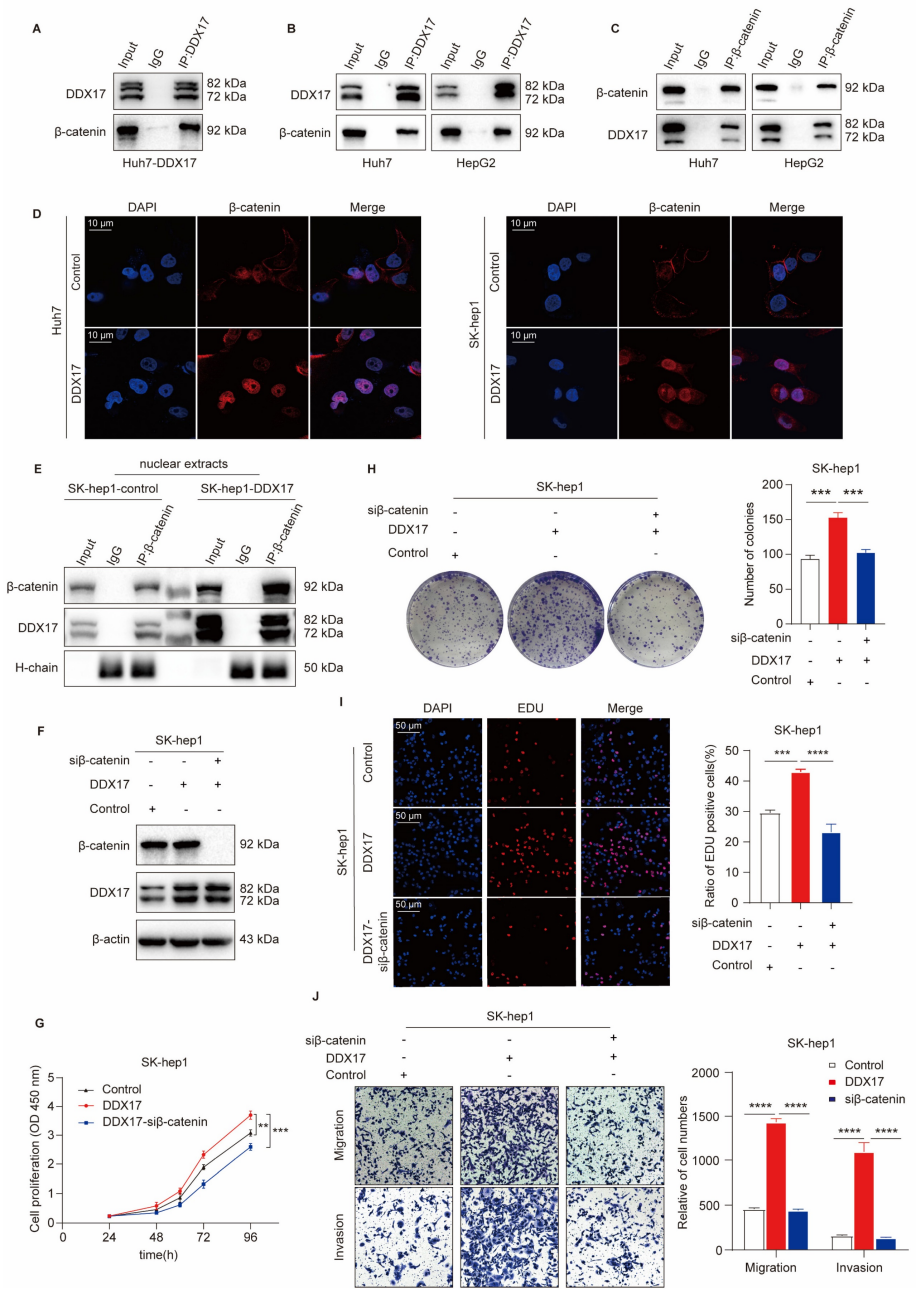
** DDX17 promotes HCC progression through mediating β-catenin nuclear translocation. (A)** Co-IP was performed with primary antibody against DDX17 in Huh-7 cells with ectopic DDX17 expression. **(B-C)** The interaction between DDX17 and β-catenin was confirmed by reciprocal IP, followed by Western blotting. **(D)** Immunofluorescence staining was conducted to determine the correlation between DDX17 expression and localization of β-catenin (Scale bars, 10 µm). **(E)** DDX17 and β-catenin protein levels in the nuclear extracts were determined by β-catenin Co-IP, followed by Western blotting. **(F)** The efficiency of DDX17 overexpression and β-catenin silencing was confirmed by Western blotting. **(G)** CCK8 assays detected the effect of β-catenin knockdown on DDX17 overexpression-enhanced cell viability (n = 3). **(H-I)** Colony formation and EdU assays detected the indicated HCC cell proliferation capacities (Scale bars, 50 µm; n = 3).** (J)** The indicated HCC cell migration and invasion were analyzed *via* Transwell and Invasion assays (n = 3). **p < 0.01, ***p < 0.001.

As a transcriptional co-factor, DDX17 essentially collaborates with several vital transcription factors to exert its biological function. A previous study revealed that DDX5, the homologous protein of DDX17, could directly interact with β-catenin to activate downstream gene transcription in colon cancer 27. More importantly, Li *et al.* have demonstrated that DDX17 could disassociate the E-cadherin/β-catenin complex and augment β-catenin target gene transcription, thus conferring gefitinib resistance in NSLCL 14. Therefore, we investigated whether DDX17 interacted with β-catenin and promoted its nuclear translocation in HCC. Overall, our results confirmed that DDX17 expression did not influence β-catenin mRNA and protein levels (Fig. S3A, B). Then, we performed an IP experiment in Huh7 cells with ectopic DDX17 expression. As shown in Fig. 3A, DDX17 physically bound with β-catenin. Furthermore, this interaction was validated in parent HepG2 and Huh7 cells with an endogenous relatively high expression of DDX17. The combination of DDX17 with β-catenin was further confirmed by reciprocal IP with primary antibodies against DDX17 or β-catenin, respectively (Fig. 3B, C).

As mentioned above, DDX17 is rarely located in the plasma membrane of the cell, mainly located in the nucleus. To assess whether the combination of DDX17 with β-catenin could mediate β-catenin activation and nuclear translocation. The IF was carried out in Hu7 and SK-hep1 cells with DDX17 overexpression. The results revealed that β-catenin was located mainly in the plasma membrane in parent cells. However, DDX17 overexpression significantly increased β-catenin nuclear accumulation (Fig. 3D). To further confirm whether β-catenin nuclear accumulation was due to interaction with DDX17, we separated the cytoplasm and nuclei protein. DDX17 overexpression or knockdown apparently influenced nuclear β-catenin protein levels (Fig. 3E; Fig. S3C). Simultaneously, the protein levels of DDX17 and β-catenin in the nucleus were markedly increased after DDX17 overexpression, indicated by β-catenin Co-IP results (Fig. 3E). These findings suggested that DDX17 overexpression promoted β-catenin nuclear accumulation and increased their binding potential. In addition, we downregulated β-catenin with RNA interference in SK-hep1-DDX17 cells and found that silencing of β-catenin significantly reversed the enhanced proliferation, migration, and invasion abilities mediated by DDX17 upregulation (Fig. 3F-J). Collectively, our findings revealed that the nuclear translocation of β-catenin mediated by DDX17 overexpression was responsible for HCC growth and metastasis.

### CXCL8, a transcriptional target of DDX17, is pivotal for DDX17-mediated HCC cell proliferation and migration

**Figure 4:**
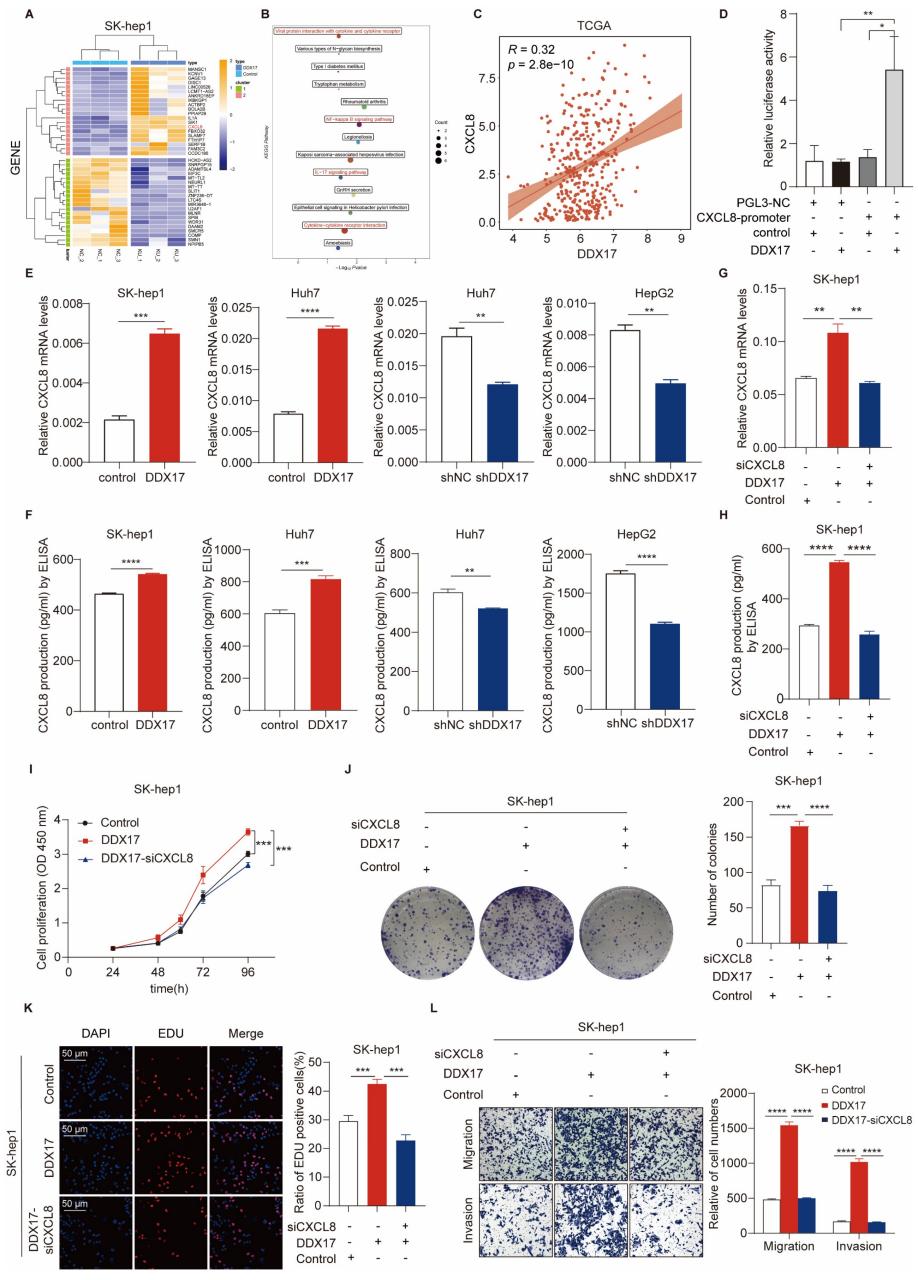
** CXCL8, a transcriptional target of DDX17, is pivotal to DDX17-mediated HCC cell proliferation and migration. (A)** A heatmap of the top 20 differentially expressed up- and down-regulated genes based on RNA-sequencing results. **(B)** KEGG pathway enrichment analysis of differentially expressed genes. **(C)** Correlations between DDX17 and CXCL8 mRNA levels were analyzed by Spearman's test using the TCGA.** (D)** Dual-luciferase reporter assay was conducted to detect relative luciferase activity of CXCL8 promoter reporter vector, after co-transfection of control or DDX17 plasmid in 293T cells. **(E-H)** mRNA and serum levels of CXCL8 were detected by RT-qPCR and ELISA after DDX17 overexpression or knockdown (n = 3). **(I)** CCK8 assay detected the influence of CXCL8 silencing on SK-hep-1 cells with DDX17 overexpression (n = 3). **(J)** Colony formation assays detected the indicated HCC cell proliferative capacities. **(K)** EdU assay detected the effect of CXCL8 knockdown on Sk-hep-1 proliferation (Scale bars, 50 µm; n = 3). **(L)** Transwell and Invasion assays were employed to analyze the indicated HCC cell migratory and invasive abilities. **p < 0.01, ***p < 0.001.

To identify the downstream regulatory genes of DDX17, we performed RNA-sequencing analysis of SK-hep1 cells with or without DDX17 overexpression. Hundreds of genes were regulated (Table S6). Among them, the top 20 genes were shown in the heatmap (Fig. 4A). The KEGG analysis revealed that “cytokine receptor activity”, “NF-kappa B signaling” and related chemokine signaling pathways were among the top pathways enriched in these genes (Fig. 4B). Combining differentially expressed genes with enriched pathways, CXCL8 was identified as the most intriguing target candidate of DDX17. In the TCGA and ICGC databases, DDX17 was positively related to the CXCL8 in the mRNA level (Fig. 4C; Fig. S4A). To further validate the transcriptional activation effect of DDX17 on CXCL8, a luciferase reporter assay was carried out. The relative luciferase activity was significantly escalated when CXCL8 promoter and DDX17 plasmids were co-transfected into HEK293T cell compared with the control (Fig. 4D). RT-qPCR and ELISA assay proved that DDX17 not only increased the mRNA expression of CXCL8, but also promoted the production of CXCL8 in the serum (Fig. 4E, F). Consistent with the results of ELISA assays, CXCL8 protein levels were significantly increased after overexpression of DDX17 and decreased after knockdown of DDX17 (Fig. S4B).

To verify whether CXCL8 is involved in DDX17-induced HCC progression, we silenced CXCL8 levels in SK-hep1 cells with DDX17 overexpression (Fig. 4G, H). Cell viability, colony formation, and EdU assays manifested that CXCL8 knockdown significantly abrogated DDX17-mediated enhanced abilities of cell proliferation (Fig. 4I-K). Meanwhile, the migration and invasion capabilities were also impaired in the CXCL8 knockdown group compared to the SK-hep1-DDX17 group (Fig. 4L). Taken together, CXCL8, which is a transcriptional target of DDX17, is responsible for DDX17-induced HCC progression.

### DDX17/β-catenin complex participates in the transcription of CXCL8 by interacting with NF-κB/p65

**Figure 5:**
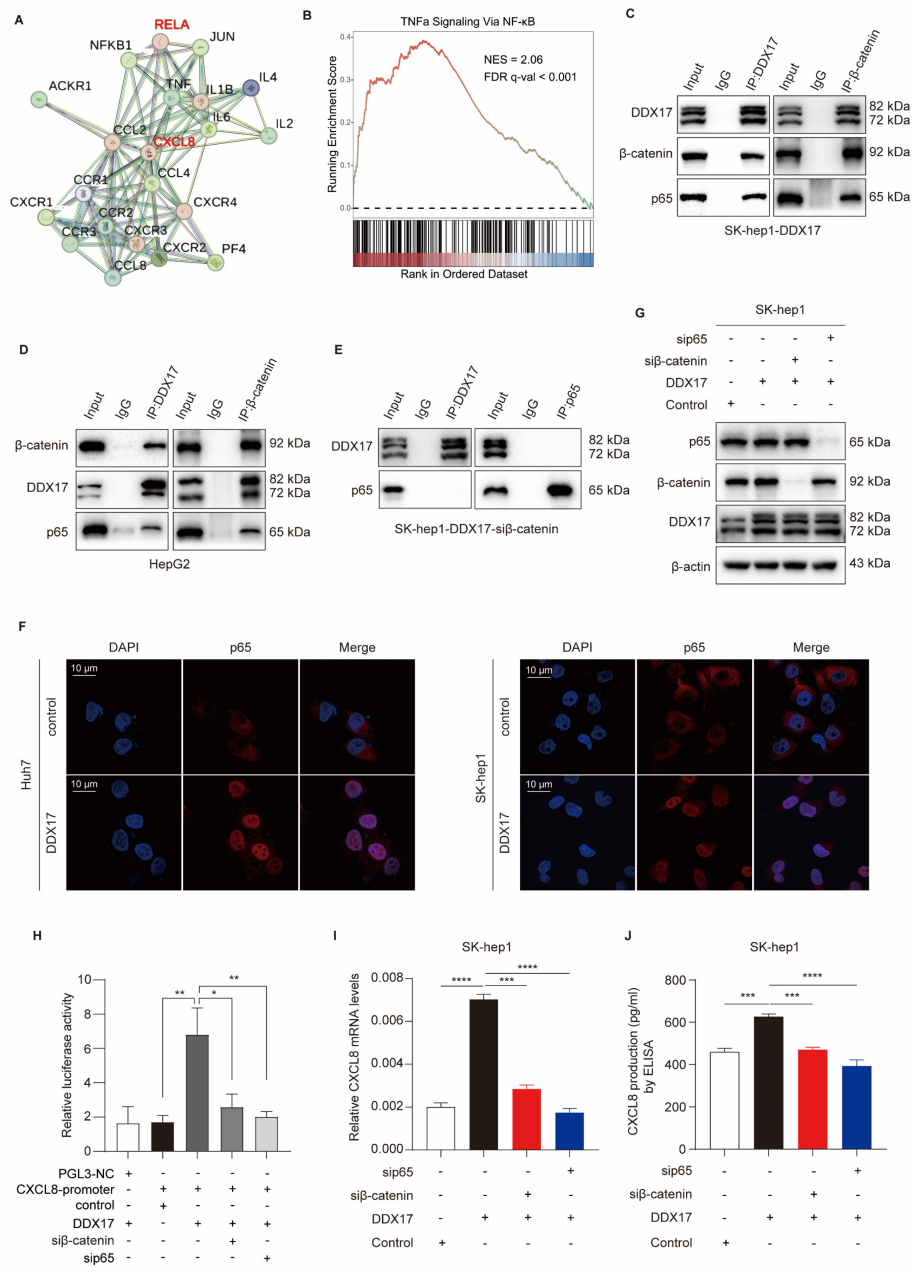
** DDX17/β-catenin complex participates in CXCL8 transcription through interaction with NF-κB/p65. (A)** The STRING bioinformatics database was deployed to analyze functional interaction between CXCL8 and NF-κB/p65.** (B)** Gene-set enrichment analysis (GSEA) plots of “TNFα Signaling via NF-κB” pathway-related signature in DDX17-overexpressed cells *vs* control cells. **(C-D)** Reciprocal Co-IP assay with primary antibodies against DDX17 or β-catenin indicated the interaction of DDX17/β-catenin/NF-κB in SK-hep1 cells with DDX17 overexpression or HepG2 cells with high endogenous expression of DDX17. **(E)** The interaction between DDX17 and NF-κB was confirmed by reciprocal Co-IP after β-catenin depletion. **(F)** IF staining inidcated NF-κB/p65 nuclear translocation after DDX17 overexpression (Scale bars, 10 µm; n = 3). **(G)** Western blotting was utilized to detect NF-κB/p65 and β-catenin protein levels after NF-κB/p65 or β-catenin knockdown.** (H)** Dual-luciferase reporter assay was employed to detect relative luciferase activity of CXCL8 promoter reporter vector, after co-transfection of control plasmid, DDX17 plasmid, β-catenin siRNA or p65 siRNA in 293T cells (n = 3). **(I-J)** CXCL8 mRNA and secretion levels were detected by RT-qPCR and ELISA after DDX17 overexpression, NF-κB/p65 or β-catenin knockdown (n = 3). *p < 0.05, **p < 0.01, ***p < 0.001.

Aiming at exploring the underlying mechanism of upregulating CXCL8 transcription, the STRING bioinformatics database ((https://string-db.org) was utilized to analyze the predicted functional partners, and NF-κB/p65 subunit was identified as the upstream target of CXCL8 (Fig. 5A). Notably, NF-kappa B signaling was also significantly enriched according to gene-set enrichment analysis (GSEA) of our transcriptional data (Fig. 5B). Extensive research has demonstrated that CXCL8 is activated by canonical NF-κB-dependent pathway, which further facilitates the progression and metastasis of various cancers 8, 28. More importantly, Lazarian *et al.* found that β-catenin physically interacted with NF-κB/p65 and participated in NF-κB-modulated the transcription of different chemokines, including IL-6, IL-1β and CXCL-8 29. These findings prompted us to verify whether the DDX17/β-catenin complex physically interacts with NF-κB and participates in NF-κB-mediated CXCL8 transcription.

As shown in Fig. 5C and D, DDX17 co-immunoprecipitated with β-catenin and p65 in HepG2 and SK-hep1 cells with high endogenous or ectopic DDX17 expression. Meanwhile, reciprocal Co-IP results also demonstrated that the DDX17 and NF-κB/p65 proteins could be pulled down by β-catenin antibody (Fig. 5C, D). Additionally, the DDX17 and β-catenin could also be co-immunoprecipitated with an antibody against NF-κB/p65 in TNF-α presence (Fig. S5A). To further confirm physical interaction between DDX17, β-catenin and RELA, we have synthesized indicated plasmids with different tags (HA-DDX17, c-Myc-β-catenin and Flag-p65), and co-transfected them in 293T cells. Co-IP results confirmed that HA antibody could pull down c-Myc-β-catenin and Flag-p65 proteins, whether HA-DDX17 was co-transfected with c-Myc-β-catenin or Flag-p65 (Fig. S5B). Moreover, we have employed the Proximity Ligation Assay (PLA) to strengthen the evidence of direct physical interaction among DDX17, β-catenin and RELA in their native cellular environment (Fig. S5C).

To explore the role of β-catenin in regulating the association between DDX17 and NF-κB, we silenced β-catenin with RNA interference. Reciprocal Co-IP results showed that any interaction between DDX17 and p65 failed to be detected, regardless of which antibody was used, indicating that β-catenin is crucial for DDX17 to recruit NF-κB/p65 (Fig. 5E; Fig. S5D).

Subsequently, IF was performed and the fluorescent intensity of p65 in the nucleus was dramatically increased due to DDX17 ectopic expression, indicating the nuclear translocation and transcriptional activity of NF-κB (Fig. 5F). To determine whether NF-κB-induced CXCL8 transcriptional activation was regulated by DDX17/β-catenin complex, we conducted a dual-luciferase assay containing a CXCL8 promoter reporter and DDX17 constructive expression plasmids in 293T cells co-transfected with β-catenin or p65 siRNA oligonucleotides. CXCL8 was transcriptionally activated after the co-transfection of DDX17 and CXCL8 gene promoter plasmids, while this activation effect was significantly inhibited by either β-catenin or p65 knockdown (Fig. 5H). Meanwhile, CXCL8 mRNA expression and serum levels were ascertained by RT-qPCR or ELISA, revealing a significant CXCL8 reduction in DDX17-overexpressing cells with β-catenin or p65 silencing (Fig. 5I, J). Altogether, our results revealed that β-catenin orchestrates the interaction between DDX17 and NF-κB/p65, which induces NF-κB-mediated transcriptional activation of CXCL8.

### CXCL8 induces DDX17/β-catenin/p65-dependent autocrine activation *via* phosphorylating NF-κB/p65

As mentioned above, we have demonstrated that CXCL8 was involved in DDX17/β-catenin/p65-mediated transcriptional activation and HCC progression. Interestingly, previous studies have reported that CXCL8/IL-8 induces NF-κB activation through increasing IκBα phosphorylation 30. These results inspired us to explore whether CXCL8 promotes NF-κB activation and nuclear translocation, which further strengthens the interaction between DDX17/β-catenin with NF-κB/p65 via a positive feedback loop.

**Figure 6:**
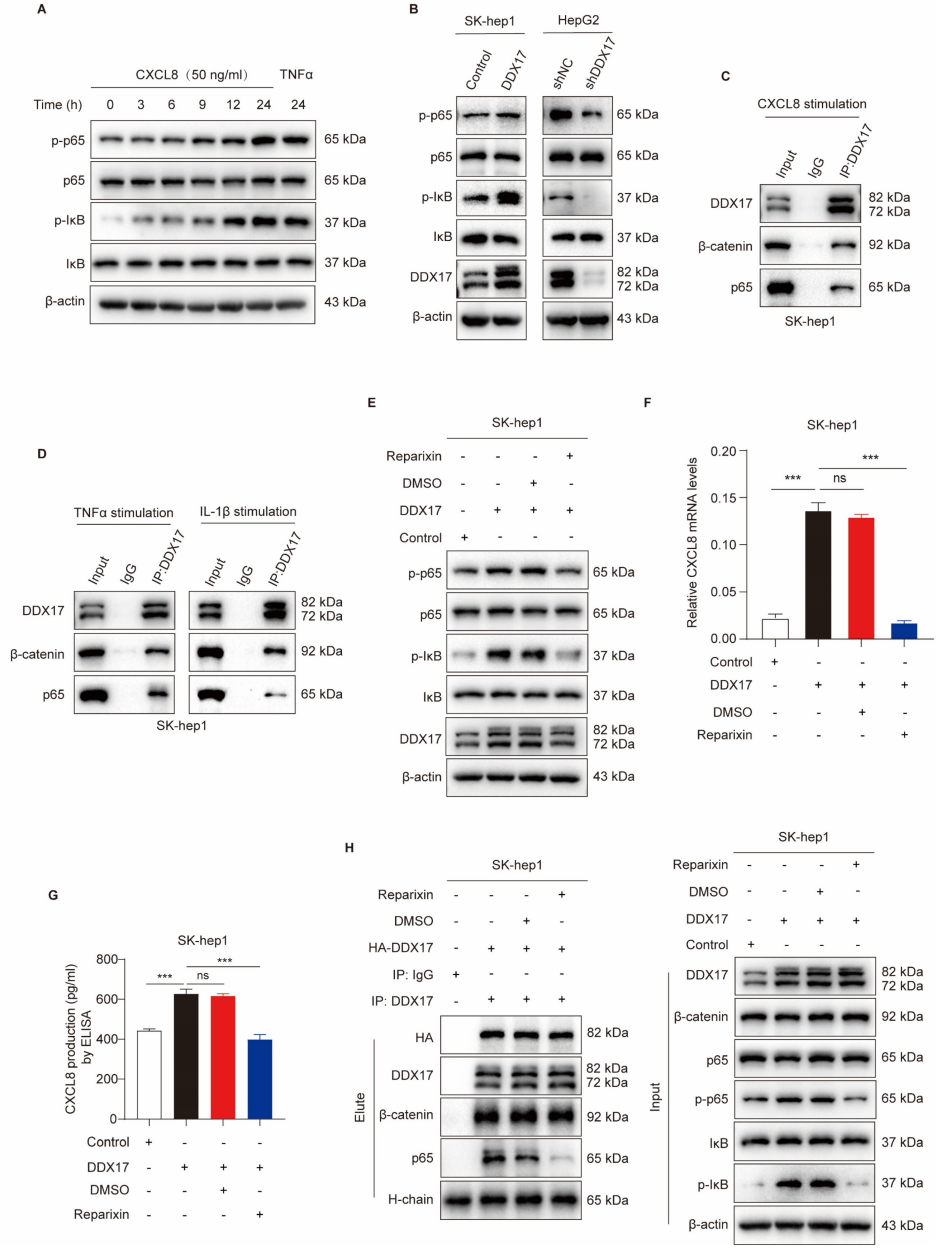
** CXCL8 induces DDX17/β-catenin/p65-dependent autocrine activation via phosphorylating NF-κB/p65. (A)** Western blotting was utilized to analyze NF-κB/p65, p-p65, IκB, and p-IκB protein levels in SK-hep1 cells after stimulated with CXCL8 or TNFα (15 ng/mL). **(B)** DDX17, NF-κB/p65, p-p65, IκB, and p-IκB protein levels were analyzed after DDX17 overexpression or knockdown. **(C)** Co-IP experiment was performed to confirm DDX17/β-catenin/NF-κB/p65 interaction in the presence of human recombinant CXCL8 (50 ng/mL, 24 h).** (D)** The interaction of DDX17/β-catenin/p65 was detected by Western blotting under TNFα (15 ng/mL, 24h) or IL-1β (10 ng/mL, 24h) stimuli.** (E)** The protein levels of NF-κB/p65, p-p65, IκB, and p-IκB were detected after DMSO or Reparixin treatment at 1 μM. **(F-G)** CXCL8 mRNA and secretion levels were detected by RT-qPCR and ELISA after DMSO or Reparixin treatment (n = 3). **(H)** Co-IP experiment was performed to validate the association of DDX17/β-catenin/p65 under DMSO or Reparixin (1 μM, 24 h) treatment. ***p < 0.001.

As described in Fig. 6A, we treated parent SK-hep1 cells with recombinant human CXCL8 ranging from 3h to 24h. Compared with the TNFα-treated group, which served as a positive control, rCXCL8 significantly escalated phosphorylated IκBα and p65 levels in a time-dependent manner. Since DDX17 augments CXCL8 gene transcription, and then elevates its serum level in a β-catenin/NF-κB-dependent manner, we detected the phosphorylation of IκBα and p65 in different HCC cells with DDX17 overexpression or knockdown. Upregulation of DDX17 in SK-hep1 cells increased the levels of phosphorylated IκBα and p65 because of the elevation of CXCL8 in the serum. Conversely, downregulation of DDX17 significantly diminished phosphorylation of IκBα and p65 in HepG2 cells (Fig. 6B). To determine whether CXCL8-induced NF-κB activation was necessary for the association between DDX17/β-catenin and NF-κB, DDX17 was co-immunoprecipitated with β-catenin and p65 in SK-hep1 cells stimulated with rCXCL8 (Fig. 6C). This result was further validated by exposing SK-hep1 cells to TNFα and IL-1β, potent NF-κB inducers that enabled retrieving DDX17/β-catenin/p65 complex (Fig. 6D).

Two transmembrane receptors, CXCR1/2, which belong to G-protein coupled rhodopsin-like seven transmembrane domain receptors, were indispensable for CXCL8-mediated signaling activation and biological function 8. Treated SK-hep1-DDX17 cells with CXCR1/2 inhibitor Reparixin dramatically reduced DDX17-induced phosphorylation of IκBα and p65 (Fig. 6E). Meanwhile, the mRNA expression and serum level of CXCL8 also significantly decreased upon Reparixin treatment in SK-hep1-DXX17 cells (Fig. 6F, G). In addition, to explore the effects of a CXCR1/2 inhibitor on NF-κB nuclear translocation and DDX17/β-catenin/p65 complex formation, Co-IP assay was carried out. Fig. 6H depicts increased interaction between DDX17, β-catenin, and NF-κB in SK-hep1 cells in response to DDX17 overexpression, whereas Reparixin treatment abrogated the association of DDX17/β-catenin/p65 complex. The inhibitory regulation of CXCR1/2 inhibitor in the DDX17/β-catenin/NF-κB complex-mediated CXCL8 transcriptional activation was further validated by an alternative inhibitor- Navarixin (Fig. S6A-D). Collectively, these data suggested that CXCL8 augmented the interaction of NF-κB with DDX17/β-catenin and enhanced its transcriptional activity in an NF-κB-dependent manner.

### CXCR1/2 inhibitor Reparixin markedly inhibits DDX17-mediated HCC growth and metastasis *in vitro* and* in vivo*

**Figure 7:**
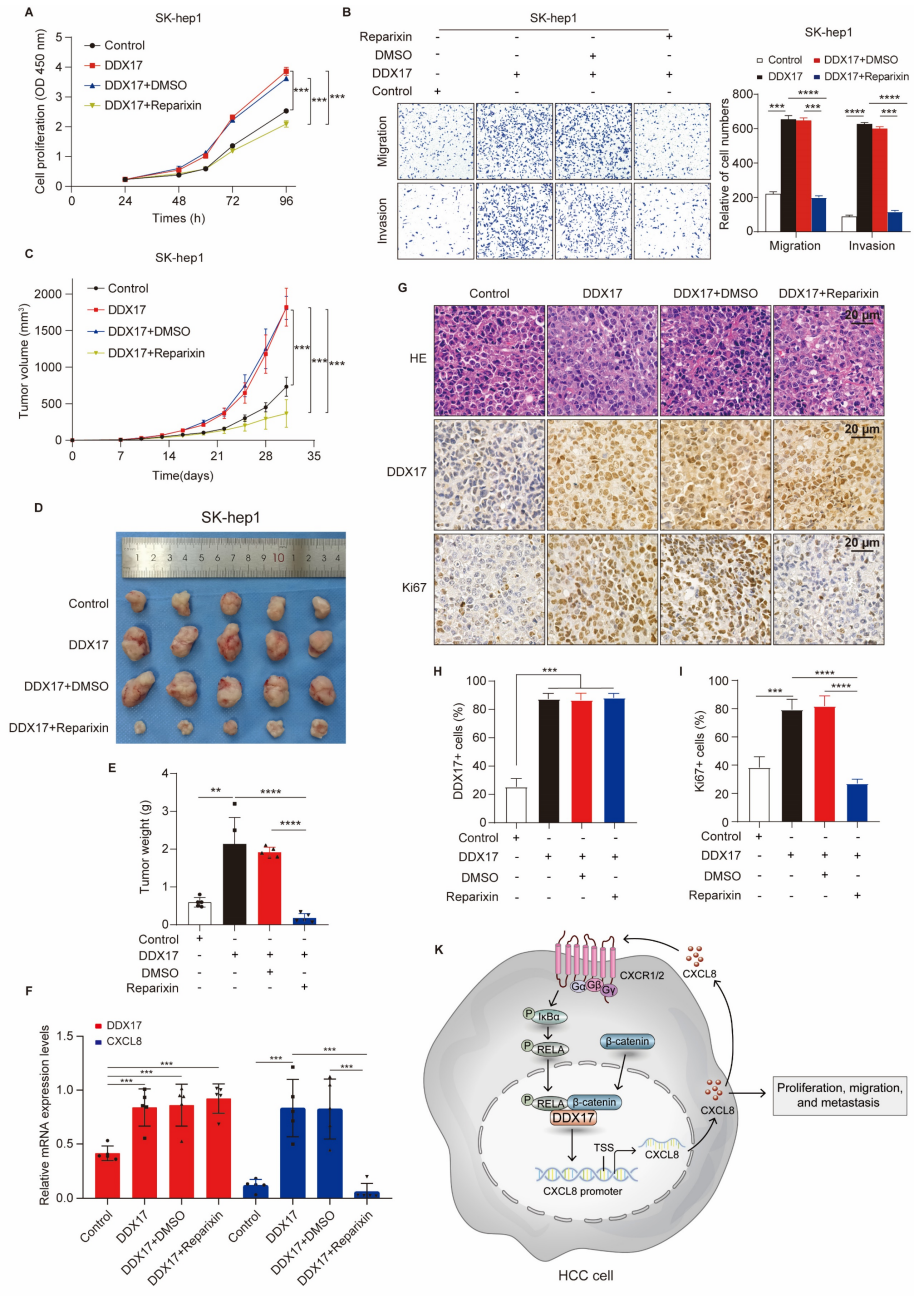
** CXCR1/2 inhibitor Reparixin markedly inhibits DDX17-mediated HCC growth and metastasis both *in vitro* and* in vivo.* (A)** CCK8 assay detected the indicated HCC cell proliferative capacities under Reparixin treatment (n = 3). **(B)** Transwell and Invasion assays analyzed SK-hep-1 cell migration and invasion with DDX17 overexpression or under Reparixin treatment (n = 3).** (C)** Tumor growth curves depict the inhibitory effect of Reparixin on DDX17-induced HCC growth *in vivo.* Two-way ANOVA with repeated measurements comparing differences between two groups (n = 5 for each group).** (D)** Xenograft tumors in nude mice were dissected and photographed at the end of the experiment. **(E)** Tumor weight of the indicated groups was measured at the end of the experiment (n = 5). **(F)** DDX17 and CXCL8 mRNA levels in xenograft tumors were measured by RT-qPCR (n = 5). **(G)** Representative images of IHC staining for Ki67 and DDX17 were presented in different xenograft groups (Scale bars, 20 µm). **(H-I)** The percentage of tumor cells positively stained with DDX17 or Ki67 was calculated in the indicated groups. **(J)** Schematic illustration of DDX17/β-catenin/NF-κB/CXCL8 feedback loop in HCC progression. **p < 0.01, ***p < 0.001.

In order to investigate the inhibitory effect of Reparixin on DDX17-mediated HCC growth and metastasis, cells with DDX17 overexpression were treated with a CXCR1/2 inhibitor. The results revealed that upregulation of DDX17 promoted HCC proliferation, migration, and invasion, whereas Reparixin treatment significantly abolished the DDX17-enhanced HCC-indicated abilities *in vitro* (Fig. 7A, B).

To further find out whether Reparixin has an inhibitory effect on DDX17-mediated HCC progression *in vivo*, xenograft mouse models were established and nude mice were randomized into four different groups. In contrast to the control, DDX17 overexpression in SK-hep1 cells promoted tumor growth, and increased tumor weight (Fig. 7C-E). However, tumor growth and weight were significantly diminished in the group of mice intraperitoneally administered with reparixin than in the DDX17-overexpressing group. RT-qPCR analysis further demonstrated that the mRNA expression of CXCL8 was decreased in tumors treated with the CXCR1/2 inhibitor (Fig. 7F). In addition, we detected the intensity of DDX17 and Ki-67 by IHC staining in the indicated groups. IHC analysis revealed that Reparixin treatment significantly inhibited the expression of Ki67, which was upregulated by DDX17 overexpression (Fig. 7G- I). Taken together, DDX17 promoted tumor growth and facilitated HCC metastasis, whereas the CXCR1/2 inhibitor, Reparixin, reversed the oncogenic effect of DDX17 in HCC progression.

## Discussion

Typically, HCC predominantly evolves from progressively worsening hepatic inflammation, yet the underlying mechanism of how inflammatory processes are regulated in the context remains poorly understood. Notably, NF-κB is virtually activated in chronic liver diseases and is crucial in governing inflammatory response in HCC *via* transactivating IL-6, CXCL8, and TNFα, among others 31. In current study, we determined a novel function of an RNA-binding protein, DDX17, which was aberrantly upregulated in HCC, in regulating the expression and secretion of CXCL8 through physical interaction with NF-κB. This study provides novel insight into how inflammation is activated in HCC pathogenesis and provides a potential therapeutic strategy for inflammation-induced HCC progression.

The DDXs family, well-characterized RNA binding proteins (RBPs) for their function in RNA biogenesis, is indispensable in tumorigenicity and metastasis as transcriptional co-activators 32. As a prototypical DDX protein, DDX17 has been demonstrated to function as a transcriptional co-factor to facilitate chromatin accessibility and transcriptional activity at target gene loci, thereby augmenting the malignant potential of various malignancies, including lung, breast and liver cancer 14, 15, 33. In particular, DDX17 was overexpressed in HCC tissues and might be presented as an oncogenic factor in HBV-related HCC tumorigenesis 21, 33. Nevertheless, the oncogenic role of DDX17 as a transcriptional co-activator in HCC remains incompletely understood, and studies were urgently needed to systematically investigate the underlying molecular mechanism in DDX17-mediated HCC progression both *in vitro* and *in vivo*.

Herein, we explored DDX17 expression in HCC tissues in comparison to adjacent normal tissues in two independent cohorts. Consistently, we demonstrated a marked elevation of DDX17 expression within HCC tissues and identified it as an unfavorable factor for prognosis in HCC patients. Furthermore, we clarified DDX17 oncogenic implications in tumor growth and metastasis *in vitro* and *in vivo*. Mechanistically, DDX17 interacted with β-catenin and strengthened its nuclear translocation. Silencing of β-catenin significantly abrogated DDX17-mediated cell growth and migration. Consistent with our findings, DDX17, which is predominantly located in the nucleus, was proven to disassociate the E-cadherin/β-catenin complex and promote β-catenin nuclear accumulation in NSCLC 14. Typically, the β-catenin activation and phosphorylation are controlled by canonical Wnt signaling. Without Wnt signaling, β-catenin is subjected to continuous phosphorylation following the cytoplasmic segment, including glycogen synthase kinase-3β (GSK-3β), AXIN, APC, and casein kinase I (CK1), which leads to its proteasomal degradation 34. Hence, our study might reveal a novel mechanism of DDX17-mediated β-catenin activation and nuclear import that is independent of Wnt signaling. However, the underlying mechanism by which DDX17 promotes β-catenin nuclear translocation remains to be further explored.

To identify the transcriptional target genes of DDX17, we performed RNA sequencing of HCC cells with ectopic DDX17 expression. Several subsets of inflammation-related pathways were significantly enriched among the top 10 KEGG pathways, including NF-κB signaling, chemokine and cytokine-cytokine receptor signaling pathways. As mentioned above, HCC is a prototypical inflammation-related cancer with aberrant activation of numerous cytokines and chemokines. Considering the significant fold change of differentially expressed genes, CXCL8 was identified as the transcriptional target of DDX17. Further experiments validated that DDX17 promoted the expression and secretion of CXCL8 through physical interaction with NF-κB. Ablation of CXCL8 or RELA significantly reversed the enhanced proliferative and migratory abilities mediated by DDX17 overexpression, indicating its crucial role in DDX17-induced HCC growth and metastasis.

Meanwhile, other pro-inflammatory chemokines were also significantly enriched after DDX17 overexpression, including CXCL1, CXCL2, IL-1α, and IL4-induced protein (Table S6). Intriguingly, a recent study has reported that DDX17 overexpression might exacerbate liver steatohepatitis and elicit the activation of M1 macrophages in murine NASH models 22. In light of these findings, our study might indicate the critical involvement of DDX17 in inflammation-related HCC proliferation and metastasis. Nevertheless, the molecular mechanism through which DDX17 contributes to the transition from NASH to HCC remains unclear. In addition, the mechanism by which DDX17-mediated inflammatory response might regulate immune cell infiltration within the TME needs to be further investigated.

To elucidate DDX17-induced CXCL8 expression, we found that DDX17 interacted with NF-κB and promoted its nuclear accumulation *via* β-catenin. Notably, a previous study has showcased that β-catenin interacts with NF-κB and participate in governing NF-κB target genes such as WNT16, IL-6, and IL-8, which contribute to β-catenin stabilization via a positive feedback loop 29. Another study also indicated that NF-κB factor RELA locally recruits DDX17 at the genomic target exons, and participats in DDX17-dependent splicing regulation 35. Our work demonstrated that silencing of β-catenin diminished the association between DDX17 and NF-κB, and simultaneously inhibited NF-κB induced CXCL8 transcriptional activation. Collectively, our study provides new insights that β-catenin mediates the interaction of DDX17/NF-κB and enhances NF-κB transcriptional activity.

CXCL8, also designated as IL-8, is one of the most renowned and intensively studied proinflammatory chemokines 36. Secreted by monocytes, macrophages, epithelial cells, and endothelial cells, CXCL8 is crucial in neutrophil chemotaxis and activation upon exposure to endogenous and exogenous pro-inflammatory stimuli 37. In cancer cells, CXCL8 integrates with multiple intracellular signaling, including PI3K/AKT, JAK/STAT, and PLC/PKC pathways, to promote tumor growth, metastasis, angiogenesis and chemoresistance through binding to the cell membrane surface receptor CXCR1/2 8, 38. Here, we demonstrated that DDX17 mediates CXCL8 overexpression, thus enhancing HCC cell proliferation and invasion. More interestingly, IκB, the NF-κB inhibitor, was regulated by CXCL8-induced signaling activation. CXCL8 stimulation significantly enhanced the phosphorylation of IκB, thus activating NF-κB and promoting its nuclear translocation. This finding was further validated by stimulation with TNFα, a canonical NF-κB activation inducer. Simultaneously, recombinant CXCL8 stimuli promoted the interaction between DDX17/β-catenin and RELA, and enhanced its transcriptional activity because of the nuclear translocation of p65. Consequently, these findings indicate the presence of a positive feedback loop involving DDX17/β-catenin/NF-κB/CXCL8 complex promotes the progression of HCC.

To explore whether the blockade of CXCL8-induced NF-κB signaling activation would inhibit DDX17-mediated HCC progression, we focused on the CXCR1/2 inhibitor. CXCR1/2, a G-protein coupled rhodopsin-like seven transmembrane domain receptors, is mainly expressed on neutrophils, macrophages, as well as cancer cells, and is indispensable for CXCL8-mediated signaling activation and biological function 39. To date, although CXCL1-8 exhibit affinities for CXCR1/2, the majority of research on CXCR1/2 inhibitors has primarily focused on the role of CXCL8 40, 41. A variety of CXCR1/2 inhibitors have been developed for the treatment of inflammation-related diseases, metabolic disorders, as well as various cancers 41. Among them, Reparixin, a small molecular antagonist against CXCR1/2, has been intensively studied for its great potential for multiple cancer treatments, including colorectal, breast, and gastric cancers 42-46. For instance, the combination of Reparixin with docetaxel has shown significant efficacy in reducing the tumor size in the models of human breast cancer cell lines and patients-derived xenografts, highlighting its therapeutic potential for breast cancer treatment 44. In our study, we found that Reparixin would disrupt the positive feedback loop that promotes HCC proliferation and cell migration via disassociating DDX17/β-catenin with NF-κB. Reparixin inhibited IκBα and NF-κB protein phosphorylation, which was mediated by DDX17-induced CXCL8 overexpression, thus impeding NF-κB nuclear translocation and interrupting DDX17/β-catenin/p65 association. Meanwhile, Reparixin treatment also abolished the HCC growth facilitated by DDX17 overexpression *in vivo*. Consequently, our study strongly indicated the potential therapeutic role of Reparixin in blocking DDX17-mediated inflammatory chemokine secretion and HCC progression. Notably, more than one clinical trial has been launched to evaluate the efficacy and safety of Reparixin in patients suffering from different cancers, including locally advanced breast cancer and myeloproliferative neoplasms (NCT 02001974, 02370238, 05835466). Our findings also implied that more clinical trials might be carried out to assess the efficacy of CXCR1/2 inhibitors in patients with HCC.

## Conclusion

The current study emphasizes the critical implication of DDX17 in HCC proliferation and metastasis and its value as an independent prognostic factor. DDX17 interacts with β-catenin and NF-κB to promote their nuclear translocation, thus transactivating the expression of CXCL8. More interestingly, CXCL8 induces the DDX17/β-catenin/NF-κB-dependent autocrine activation via phosphorylating IκB and NF-κB proteins. Furthermore, interrupting the association between DDX17/β-catenin and NF-κB with CXCR1/2 inhibitor would markedly reverse DDX17-mediated HCC proliferation and metastasis. Our study offers novel perspectives into DDX17's oncogenic role in HCC progression, and the applicability of CXCR1/2 inhibitors to block DDX17-mediated CXCL8 signaling activation might be a potential strategy for HCC treatment.

## Supplementary Material

Supplementary figures and tables 1-5.

Supplementary table 6.

## Figures and Tables

**Table 1:** Correlation between DDX17 expression and clinicopathological characteristic of 88 cases of HCC patients

Characteristics	Total	DDX17 expression		*P* value
		High	Low	
Age^a^				
≤ 50 years	30	7	23	0.002**
> 50 years	57	33	24	
Gender				
Male	78	37	41	0.297
Female	10	3	7	
Cirrrhosis				
Presence	31	13	18	0.66
Absence	57	27	30	
Maximal size^a^				
≤ 5 cm	40	19	21	0.625
> 5 cm	47	20	27	
Tumor number^a^				
Single	65	27	38	0.195
Multiple	15	9	6	
Histological stage				
Stage Ⅰ-Ⅱ	54	20	34	0.046*
Stage Ⅲ-Ⅳ	34	20	14	
TNM stage^a^				
Stage Ⅰ-Ⅱ	41	20	24	0.269
Stage 3-4	41	20	19	
Ki67 staining				
< 30% (negative)	70	28	42	0.043*
≥ 30% (positive)	18	12	6	
P53 staining				
< 50% (negative)	36	20	16	0.113
≥ 50% (positive)	52	20	32	
PD-L1 staining^a^				
< 5% (negative)	27	7	20	0.008*
≥ 5% (positive)	39	23	16	

^a^ Some data are not available. *P*-value < 0.05 are in bold

**Table 2:** Univariate and multivariate analysis of factors associated with survival in 88 cases of HCC patients

Variables	Univariate analysis		
	HR	95% CI	P value
DDX17 expression (high versus low)	1.773	1.050-2.994	0.032*
Gender (male versus female)	1.509	0.602-3.780	0.380
Age (> 50 years versus ≤ 50 years)	1.093	0.625-1.913	0.754
Cirrrhosis (presence versus absence)	1.080	0.630-1.853	0.779
Tumor size (>5cm versus ≤ 5cm)	1.919	1.111-3.315	0.02*
Tumor number (multiple versus single)	1.663	0.884-3.127	0.114
Histological stage (Ⅲ-Ⅳ versus Ⅰ-Ⅱ)	1.317	0.778-2.231	0.305
TNM stage (3-4 versus 1-2)	2.616	1.465-4.670	0.001***
Ki67 staining (≥ 30% versus < 30%)	2.364	1.304-4.2833	0.005**
P53 staining (≥ 50% versus < 50%)	1.218	0.714-2.079	0.469
PD-L1 staining (≥ 5% versus < 5%)	1.155	0.616-2.167	0.653
Variables	Multivariate analysis		
	HR	95% CI	P value
DDX17 expression (high versus low)	1.898	1.066-3.378	0.030*
Tumor size (>5cm versus ≤ 5cm)	1.325	0.633-2.773	0.455
TNM stage (3-4 versus 1-2)	2.107	1.007-4.408	0.048*
Ki67 staining (≥ 30% versus < 30%)	2.110	1.129-3.944	0.019*

* *P*-value < 0.05 are in bold

## Abbreviations

AKT: Protein Kinase B
ATCC: American Type Culture Collection
CK1: Casein kinase I
CXCL: C-X-C motif chemokine ligand
CXCR: C-X-C motif chemokine receptor
DDX17: DEAD box RNA helicase 17
GSK-3β: Glycogen synthase kinase-3β
GSEA: Gene-set enrichment analysis
HCC: Hepatocellular carcinoma
IF: Immunofluorescence
IHC: Immunohistochemistry
IKK: NF-kappa-B inhibitor alpha kinase
ILs: Interleukins
IP: Immunoprecipitation
JAK: Janus kinase
KEGG: Kyoto Encyclopedia of Genes and Genomes
NASH: Non-alcoholic steatohepatitis
NF-κB: Nuclear factor kappaB
PI3K: Phosphoinositide 3-kinase
PKC: Protein Kinase C
PLC: Phospholipase C
RBPs: RNA binding proteins
siRNA: Small interfering RNA
SOX2: Sex-determining region Y (SRY)-box binding protein-2
STAT: Signal transducer and activator of transcription
STR: Short tandem repeats
TCGA: The Cancer Genome Atlas database
TNFα: Tumor necrosis factor alpha
