# Supplementary material for: DDX17-Mediated Upregulation of CXCL8 Promotes Hepatocellular Carcinoma Progression via Co-activating β-catenin/NF-κB Complex
Source: Int J Biol Sci. 2025 Jan 20;21(3):1342–60. doi: 10.7150/ijbs.104165 (PMC11781183; doi:10.7150/ijbs.104165)
Supplement: Supplementary file 1 — Supplementary figures and tables 1-5. [file ijbsv21p1342s1.pdf]

Supplementary figure legends

Figure S1. DDX17 is significantly upregulated and correlated with poor survival in an independent ESCC cohort. (A) Representative micrographs of immunohistochemical staining for DDX17 in different pathological stages of HCC tissues and adjacent normal tissues. Scale bars, 200 µm (upper), 50 µm (lower). **(B)** Immunohistochemical scores of ESCC (n = 92) and adjacent normal tissues (n = 68) were calculated by Wilcoxon's matched-pairs signed-rank test. **(C)** Kaplan-Meier analysis was used to evaluate the causal relationship between DDX17 expression and overall survival in 92 ESCC patients. **(D)** The correlation between clinicopathological factors and overall survival was evaluated by Univariate Cox regression analysis in the ESCC cohort.

Figure S2. DDX17 promotes the proliferation of Huh7 cells. (A-B) RT-qPCR was performed to determine the efficiency of DDX17 overexpression or knockdown in HCC cell lines (n = 3). **(C)** EdU assays showed the proliferative abilities of Huh7 cells after DDX17 overexpression or knockdown (Scale bars, 50 µm; n = 3). $^{**}p < 0.01$, $^{***}p < 0.001$.

Figure S3. DDX17 overexpression promotes the nuclear accumulation of β-catenin. (A) Western blot was performed to detect the protein expression of β-catenin in HCC cell lines with DDX17 overexpression or knockdown. **(B)** RT-qPCR was used to detect the mRNA expression of β-catenin in HCC cell lines that overexpressed or knocked down DDX17 (n = 3). **(C)** The protein levels of β-catenin in the nuclei were determined by Western blot assay after cytoplasmic and nuclear protein separation. Lamin A/C was used as nuclear biomarkers (n = 3).

Figure S4. DDX17 promotes the expression of the CXCL8. (A) DDX17 positively correlates with CXCL8 in ICGC database. **(B)** The protein levels of CXCL8 after DDX17 overexpression or knockdown were indicated by Western blotting.

Figure S5. DDX17 physically interacts with NF-κB through β-catenin. (A) NF-κB co-immunoprecipitated with DDX17 and β-catenin, followed with Western blot assay in the presence of TNFα stimulus (15 ng/mL, 24h). **(B)** DDX17, β-catenin and RELA plasmids were constructed with different tags and co-transfected into 293T cells, HA-DDX17 co-immunoprecipitated with c-Myc-β-catenin or Flag-p65, followed with Western blot assay. **(C)** Proximity Ligation Assay (PLA) was conducted to detect the physical interaction among DDX17, β-catenin, and RELA. Images at different magnification levels were presented. DDX17/DDX17 group was used as the positive control, while DDX17/IgG or DDX17 group acted as the negative control. **(D)** DDX17 co-immunoprecipitated with NF-κB in HepG2 cells after β-catenin knockdown, followed with Western blot assay.

Figure S6. Navarixin inhibits the interaction of DDX17/β-catenin/RELA complex and suppresses their transcriptional activity at the CXCL8 promoter. (A) The protein levels of NF-κB/p65, p-p65, IκB, p-IκB were detected after cells were treated with DMSO or Navarixin. **(B-C)** The mRNA and secretion levels of CXCL8 were detected by RT-qPCR and ELISA after DMSO or Navarixin treatment (n = 3). **(D)** Co-IP experiment was performed to validate the association of DDX17/β-catenin/p65 under DMSO or Navarixin (25 μM, 24h) treatment. ***$p < 0.001$.

Figure S1

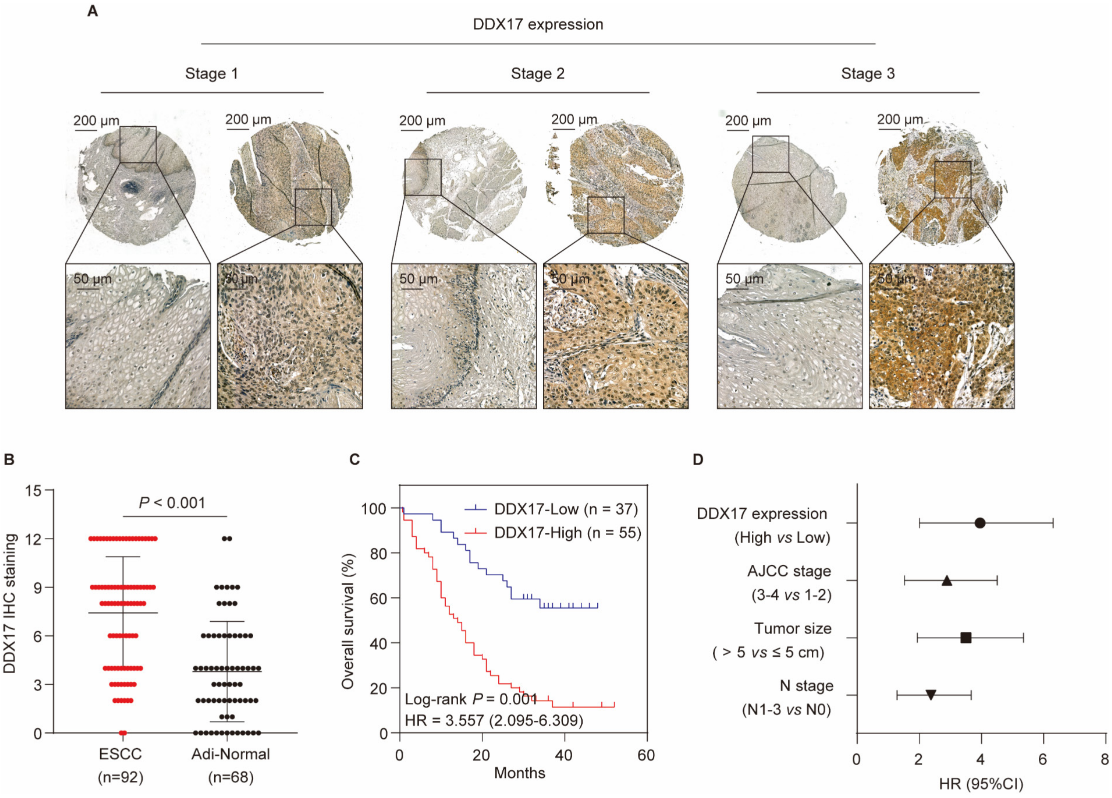
A
DDX17 expression
Stage 1
Stage 2
Stage 3
200 μm
50 μm
B
P < 0.001
DDX17 IHC staining
15
12
9
6
3
0
ESCC
(n=92)
Adi-Normal
(n=68)
C
DDX17-Low (n = 37)
DDX17-High (n = 55)
Overall survival (%)
100
80
60
40
20
0
Log-rank P = 0.001
HR = 3.557 (2.095-6.309)
0
20
40
60
Months
D
DDX17 expression
(High vs Low)
AJCC stage
(3-4 vs 1-2)
Tumor size
(> 5 vs ≤ 5 cm)
N stage
(N1-3 vs N0)
0
2
4
6
8
HR (95%CI)

1 # Figure S2

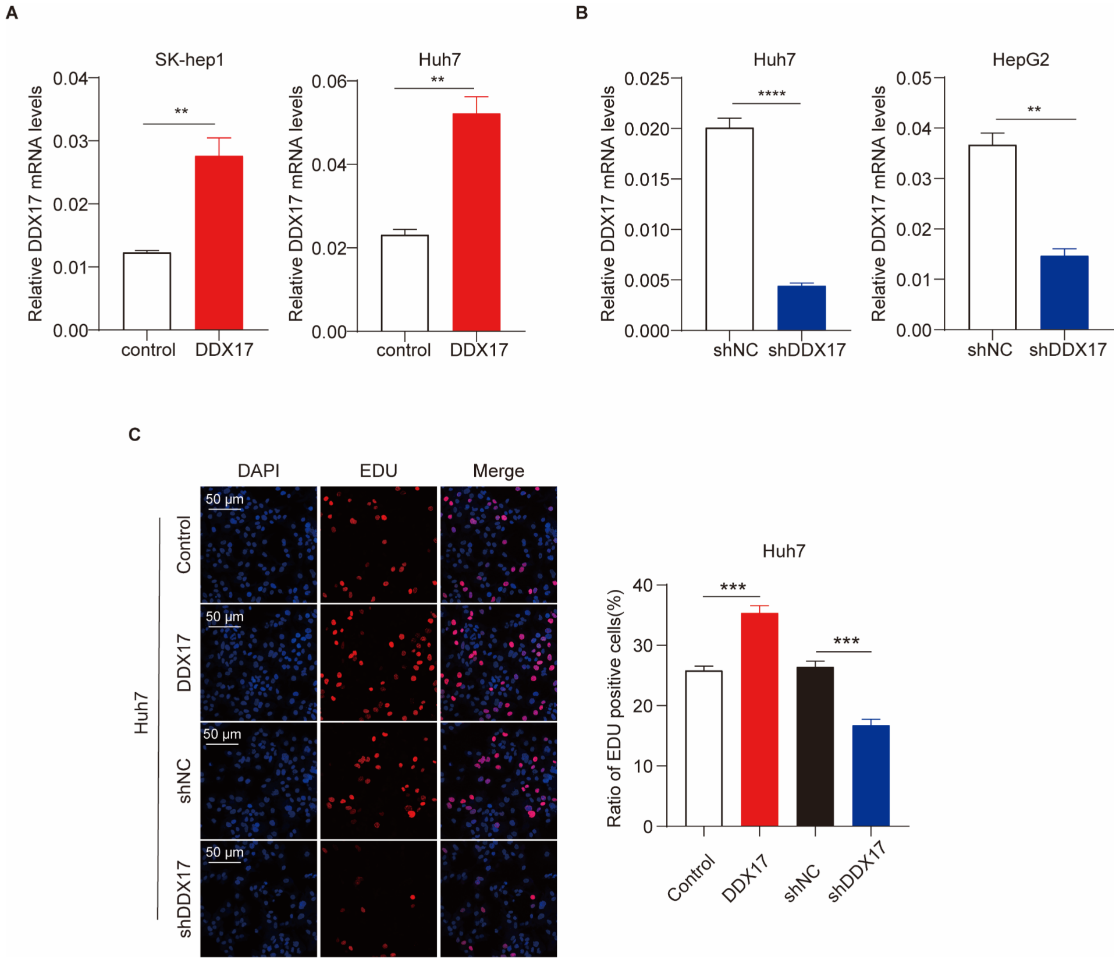
A
SK-hep1
Relative DDX17 mRNA levels
control
DDX17
Huh7
B
Huh7
HepG2
shNC
shDDX17
C
DAPI
EDU
Merge
50 μm
Control
DDX17
shNC
shDDX17
Huh7
Ratio of EDU positive cells(%)

2

1 # Figure S3

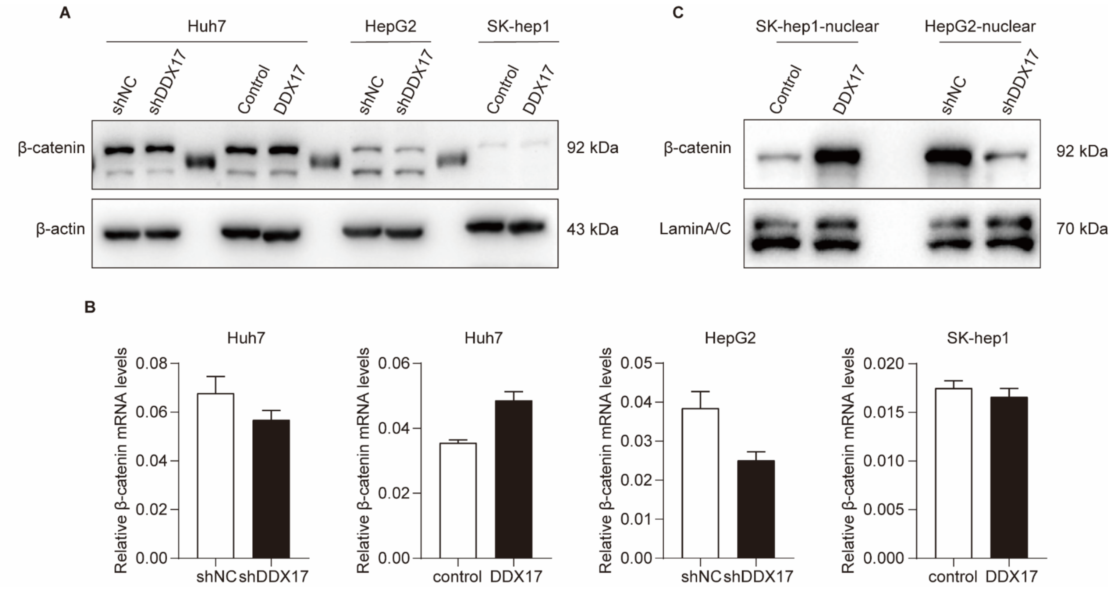

A
Huh7
HepG2
SK-hep1
shNC
shDDX17
Control
DDX17
β-catenin
β-actin
92 kDa
43 kDa
C
SK-hep1-nuclear
HepG2-nuclear
LaminA/C
70 kDa
B
Relative β-catenin mRNA levels
control

2

Figure S4

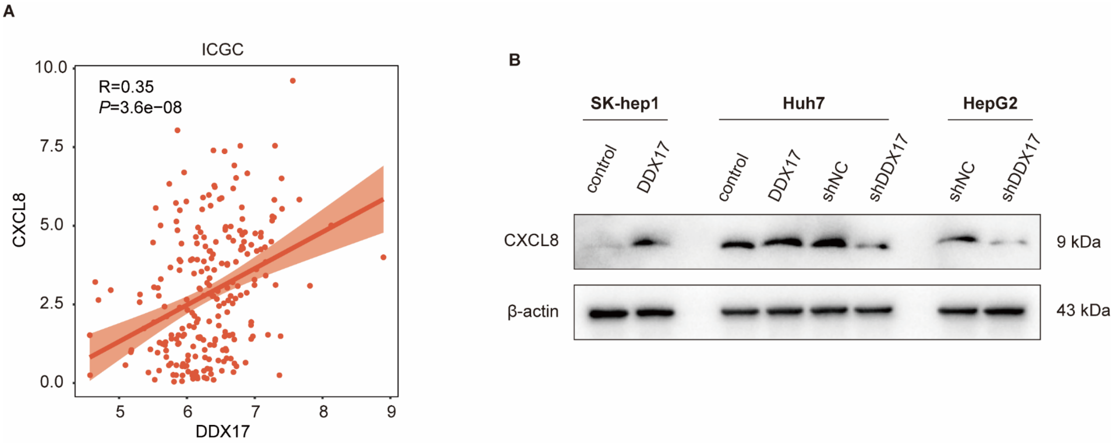
A
ICGC
R=0.35
P=3.6e−08
CXCL8
DDX17
10.0
7.5
5.0
2.5
0.0
5
6
7
8
9
B
SK-hep1
Huh7
HepG2
control
DDX17
control
DDX17
shNC
shDDX17
shNC
shDDX17
CXCL8
β-actin
9 kDa
43 kDa

Figure S5

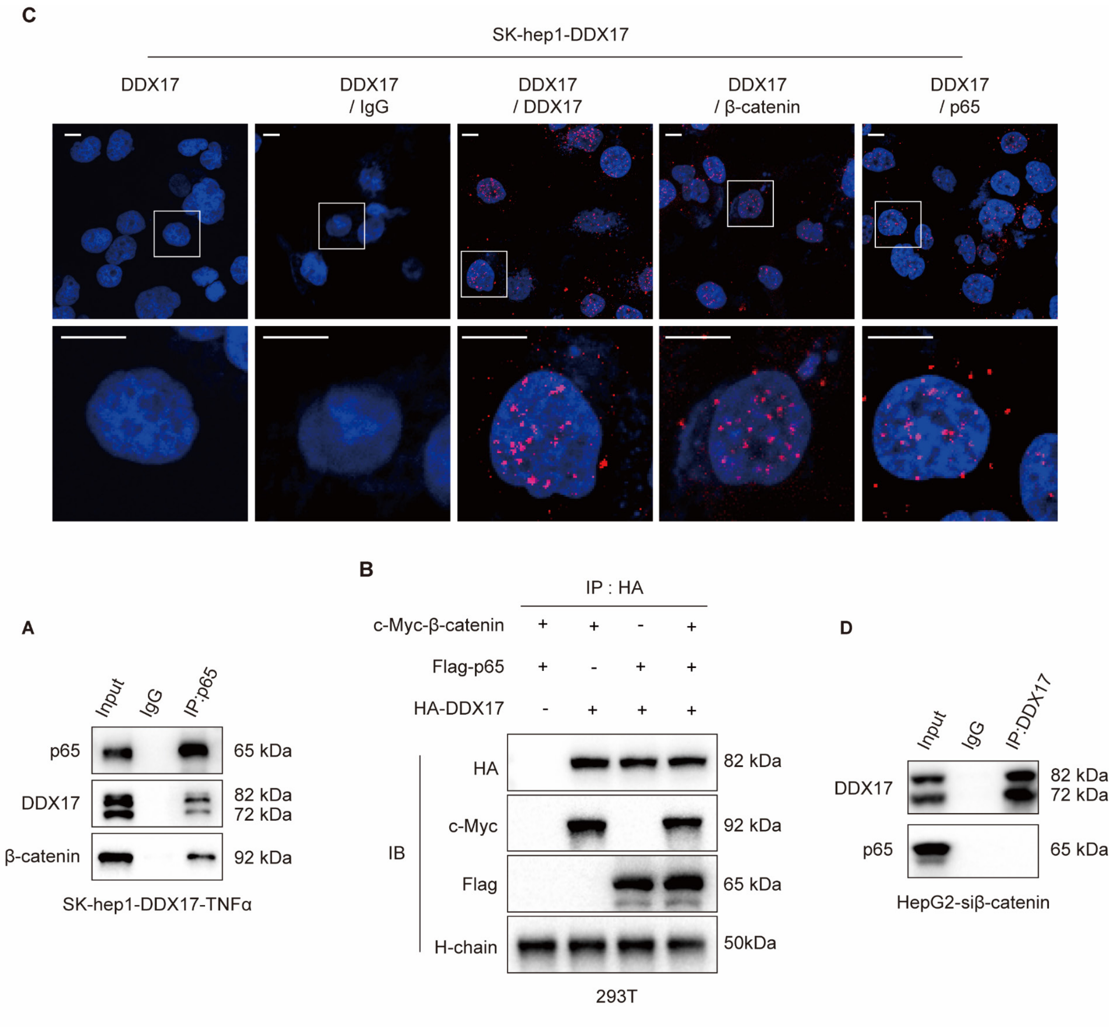
C
SK-hep1-DDX17
DDX17
DDX17 / IgG
DDX17 / DDX17
DDX17 / β-catenin
DDX17 / p65
A
Input
IgG
IP:p65
p65
65 kDa
DDX17
82 kDa
72 kDa
β-catenin
92 kDa
SK-hep1-DDX17-TNFα
B
IP : HA
c-Myc-β-catenin + + - +
Flag-p65 + - + +
HA-DDX17 - + + +
IB
HA
82 kDa
c-Myc
92 kDa
Flag
65 kDa
H-chain
50kDa
293T
D
Input
IgG
IP:DDX17
DDX17
82 kDa
72 kDa
p65
65 kDa
HepG2-siβ-catenin

Figure S6

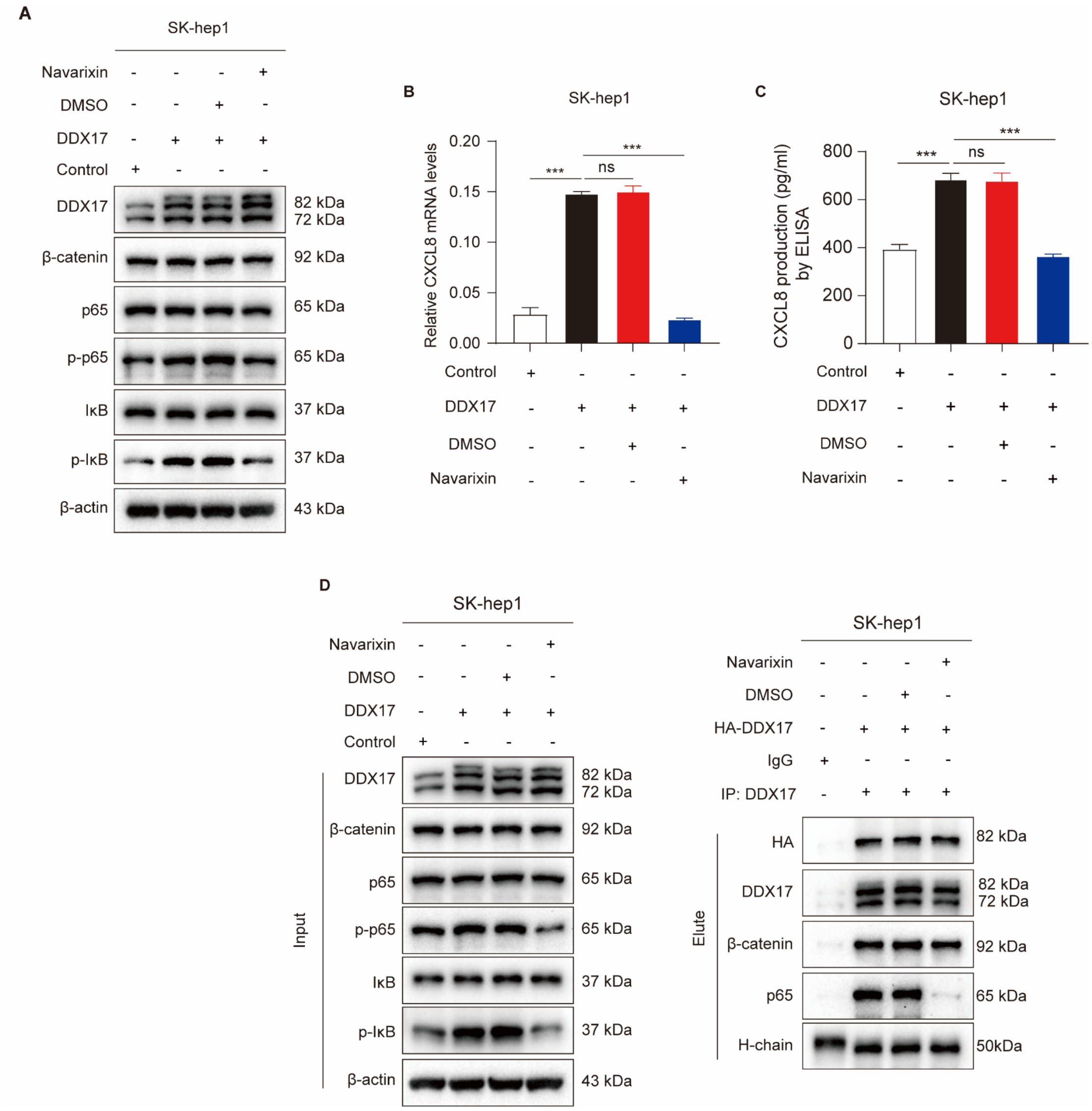
A
SK-hep1
Navarixin
DMSO
DDX17
Control
DDX17
β-catenin
p65
p-p65
IκB
p-IκB
β-actin
82 kDa
72 kDa
92 kDa
65 kDa
37 kDa
43 kDa
B
SK-hep1
Relative CXCL8 mRNA levels
0.00
0.05
0.10
0.15
0.20
***
ns
C
SK-hep1
CXCL8 production (pg/ml) by ELISA
0
200
400
600
800
D
SK-hep1
Input
HA-DDX17
IgG
IP: DDX17
Elute
HA
H-chain
50kDa

Table S1

siRNA sequences used in the study

siRNA	Sequence（5’-3’）
β-catenin-siRNA 1-sense	GCAGCUGGAAUUCUUUCUATT
β-catenin-siRNA 1-antisense	UAGAAAGAAUUCCAGCUGCTT
β-catenin-siRNA 2-sense	GGACACAGCAGCAAUUUGUTT
β-catenin-siRNA 2-antisense	ACAAAUUGCUGCUGUGUCCTT
CXCL8-siRNA 1-sense	GCCAAGGAGUGCUAAAGAATT
CXCL8-siRNA 1-antisense	UUCUUUAGCACUCCUUGGCTT
CXCL8-siRNA 2-sense	GAAGAGGGCUGAGAAUUCATT
CXCL8-siRNA 2-antisense	UGAAUUCUCAGCCCUCUUCTT
p65-siRNA 1-sense	CUUCCAAGUUCCUAUAGAATT
p65-siRNA 1-antisense	UUCUAUAGGAACUUGGAAGTT
p65-siRNA 2-sense	GCACCAUCAACUAUGAUGATT
p65-siRNA 2-antisense	UCAUCAUAGUUGAUGGUGCTT

Table S2

Primer sequences used in the study

Primer name	Primer sequences
DDX17-forward	5'-ACTGATGCAGCTTGTGGACCAC-3'
DDX17-reverse	5'-AAGCCTTCGGTCACACTCATCC-3'
CXCL8-forward	5'-TGTTAAATCTGGCAACCCTAGTCT-3'
CXCL8-reverse	5'-CTGTGAGGTAAGATGGTGGCTAA-3'
β-catenin-forward	5'-TACCTCCCAAGTCCTGTATGAG-3'
β-catenin-reverse	5'-TGAGCAGCATCAAACTGTGTAG-3'
β-actin-forward	5'-CATGTACGTTGCTATCCAGGC-3'
β-actin-reverse	5'-CTCCTTAATGTCACGCACGAT-3'

Table S3

Anti-bodies used in the study

Anti-bodies	Source
anti-DDX17	Abcam, ab180190
anti-DDX17	Biolegend, 657302
anti-β-catenin	Cell Signaling Technology, #8480
anti-p65	Abmart, T55034M
anti-p-p65(Ser536)	Abmart, TP56372S
anti-IKB	Abmart, T55026
anti-p-IKB	Abmart, T56280
anti-CXCL8	Abmart, T58948
anti-β-actin	Proteintech, 60008-1-Ig

Table S4

Table S4 Correlation between DDX17 expression levels and clinicopathological characteristic in 92 cases of ESCC

Characteristics	Total	DDX17 expression		*P* value
		High	Low	
**Gender**				
Male	75	48	27	0.104
Female	17	7	10	
**Age (years)**[a]				
> 55	79	47	32	0.758
≤ 55	12	8	4	
**Maximal size (cm)**				
> 5cm	31	22	9	0.177
≤ 5cm	61	33	28	
**Histological grade**				
I	23	15	8	0.628
II-III	69	40	29	
**T stage**				
T1-2	12	8	4	0.756
T3-4	80	47	33	
**N stage**				
N0	40	16	24	**0.001***
N1-3	52	39	13	
**M stage**				
M0	90	53	37	0.514
M1	2	2	0	
**AJCC stage**				
Stage 1-2	39	17	22	**0.009***
Stage 3-4	53	38	15	

[a] One sample was missed. *P*-value < 0.05 are in bold

Table S5

Table S5 Univariate and multivariate analysis of factors associated with survival in 92 cases of ESCC patients

Variables	Univariate analysis		
	HR	95% CI	P value
DDX17 expression (high versus low)	3.557	2.005-6.309	**0.001*****
Gender (male versus female)	1.831	0.902-3.715	0.094
Age (> 55 years versus ≤ 55 years)	0.519	0.262-1.027	0.059
Tumor size (> 5 cm versus ≤ 5 cm)	3.219	1.936-5.354	**0.001*****
Histological grade (II-III versus I)	1.122	0.629-2.002	0.697
T stage (T3-4 versus T1-2)	1.501	0.684-3.293	0.311
N stage (N1-3 versus N0)	2.17	1.283-3.669	0.004**
M stage (M1 verus M0)	2.496	0.605-10.301	0.206
AJCC stage (3-4 versus 1-2)	2.625	1.526-4.513	**0.001****
Variables	Multivariate analysis		
	HR	95% CI	P value
DDX17 expression (high versus low)	3.274	1.794-5.977	**0.001****
Tumor size (> 5 cm versus ≤ 5 cm)	3.586	2.096-6.135	**0.001*****
N stage (N1-3 versus N0)	0.755	0.315-1.808	0.528
AJCC stage (3-4 versus 1-2)	2.786	1.138-6.819	**0.025***
